# Acetylcholine Neurons Become Cholinergic during Three Time Windows in the Developing Mouse Brain

**DOI:** 10.1523/ENEURO.0542-23.2024

**Published:** 2024-07-11

**Authors:** Rene Oliver Goral, Patricia W. Lamb, Jerrel L. Yakel

**Affiliations:** ^1^Neurobiology Laboratory, National Institute of Environmental Health Sciences, National Institutes of Health, Department of Health and Human Services, Research Triangle Park, North Carolina 27709; ^2^Center on Compulsive Behaviors, National Institutes of Health, Bethesda, Maryland 20892

**Keywords:** brain development, cholinergic neurons, transgenic mice, whole-brain imaging

## Abstract

Acetylcholine (ACh) neurons in the central nervous system are required for the coordination of neural network activity during higher brain functions, such as attention, learning, and memory, as well as locomotion. Disturbed cholinergic signaling has been described in many neurodevelopmental and neurodegenerative disorders. Furthermore, cotransmission of other signaling molecules, such as glutamate and GABA, with ACh has been associated with essential roles in brain function or disease. However, it is unknown when ACh neurons become cholinergic during development. Thus, understanding the timeline of how the cholinergic system develops and becomes active in the healthy brain is a crucial part of understanding brain development. To study this, we used transgenic mice to selectively label ACh neurons with tdTomato. We imaged serial sectioned brains and generated whole-brain reconstructions at different time points during pre- and postnatal development. We found three crucial time windows—two in the prenatal and one in the postnatal brain—during which most ACh neuron populations become cholinergic in the brain. We also found that cholinergic gene expression is initiated in cortical ACh interneurons, while the cerebral cortex is innervated by cholinergic projection neurons from the basal forebrain. Taken together, we show that ACh neuron populations are present and become cholinergic before postnatal day 12, which is the onset of major sensory processes, such as hearing and vision. We conclude that the birth of ACh neurons and initiation of cholinergic gene expression are temporally separated during development but highly coordinated by brain anatomical structure.

## Graphical abstract

**Figure:**
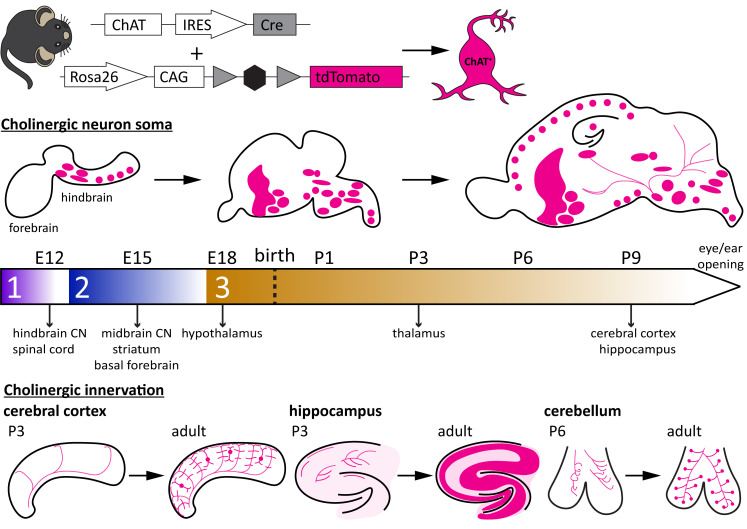
Visual Overview

## Significance Statement

Acetylcholine (ACh) neurons are required for higher brain functions and locomotion. Disturbed cholinergic signaling was observed in neurodevelopmental disorders and intellectual disability. While the role of ACh release in neural circuit function is well understood, it is unknown when ACh neurons become cholinergic. We investigated when ACh neurons become cholinergic in the developing brain. Here, we show that ACh neurons become cholinergic during three time windows pre- and postnatally. ACh neurons become cholinergic along the caudorostral direction of brain formation. In cortex and hippocampus, activation of cholinergic genes in ACh interneurons coincides with cholinergic innervation from the basal forebrain. We highlight that most ACh neurons are cholinergic before P12, the onset of major sensory functions, such as hearing and vision.

## Introduction

Acetylcholine (ACh) neurons or their projections can be virtually found in every part of the central nervous system (Ananth et al., 2023). ACh neurons are among the first neurons to be born and to arrive at their destinations and nAChRs drive other metabotropic systems from an early point in development (Role, 1996; Dwyer et al., 2008; Allaway et al., 2020). While ACh neuron involvement in neural circuit formation remains debated but unclear, altered ACh neuron markers and firing patterns have been found in many brain disorders, such as Rett syndrome, Tourette syndrome (TS), or epilepsy (Janiesch et al., 2011; Oginsky et al., 2014; Lennington et al., 2016; Bekenstein et al., 2017; Soares et al., 2017; Lozovaya et al., 2023). ACh neurons form synapses on a plethora of neurons and provide accurately timed modulation of neuronal firing which is commonly accepted as contributing to oscillatory events in various brain regions (Marrosu, 1995; Hasselmo and McGaughy, 2004; Gu and Yakel, 2011; Unal et al., 2015; Gu et al., 2017; Gu et al., 2020).

Cholinergic signaling in the central nervous system is not only required for higher brain functions/cognition, such as attention, learning, and memory, but also for pattern generation in spinal locomotor networks for muscle activity (Ballinger et al., 2016; Mille et al., 2021). Interestingly, cholinergic signaling is not restricted to neurons but is also required for glia or immune cell functions (Pabst et al., 2016; Li et al., 2018; Broide et al., 2019; Fontana et al., 2023). As a ligand, ACh targets both ionotropic nicotinic ACh receptors (nAChRs) and metabotropic muscarinic ACh receptors (mAChRs, Ballinger et al., 2016). In addition to effects on neuron firing and synaptic strength, the cholinergic system modulates neuron morphology and can directly affect the release of neuromodulators, such as dopamine (DA) and norepinephrine from axonal varicosities (Lozada et al., 2012; Morley and Mervis, 2013; Cools and Arnsten, 2022; Liu et al., 2022; Steinecke, 2022).[Table T1][Table T2]

**Table 1. T1:** Antibodies used for immunohistochemistry

Target	Donor species	Dilution	Vendor	Conjugate	Catalog #	RRID
ChAT	Goat	1:100	Millipore	N/A	AB144P-200UL	RRID:AB_90661
DsRed	Rabbit	1:500	Rockland	N/A	600-401-379	RRID:AB_2209751
NeuN	Rabbit	1:500	Abcam	N/A	ab177487	RRID:AB_2532109
Goat IgG	Donkey	1:500	Jackson ImmunoResearch	Alexa Fluor 647	705-605-147	RRID:AB_2340437
Rabbit IgG	Goat	1:500	Thermo Fisher Scientific	Alexa Fluor Plus 647	A32733	RRID:AB_2633282

10.1523/ENEURO.0542-23.2024.t1-1 Table 1-1 Animals used for experiments depicted in different figures. Download Table 1-1, DOC file.

**Table 2. T2:** Time points at which ACh neurons are cholinergic in the mouse brain in brain anatomical structures

Brain region	E12 (*n* = 3)	E15^a^ (*n* = 3)	E18^a^ (*n* = 5)	P1 (*n* = 3)	P3 (*n* = 3)	P6 (*n* = 3)	P9 (*n* = 3)	P12 (*n* = 3)	P50 (*n* = 3)	>1 year (*n* = 3)
Spinal cord	++	++	++	nd	nd	nd	nd	nd	nd	nd
Hindbrain CN	++	++	++	++	++	++	++	++	++	++
Midbrain CN	−	++	++	++	++	++	++	++	++	++
Other hindbrain nuclei	−	+	++	++	++	++	++	++	++	++
Other midbrain nuclei	−	+	++	++	++	++	++	++	++	++
Striatum	−	+^b^	++	++	++	++	++	++	++	++
Basal Forebrain	−	+	++	++	++	++	++	++	++	++
Hypothalamus	−	−	+	+	++	++	++	++	++	++
Thalamus	−	−	−	+^c^	+	++	++	++	++	++
Cerebellum^d^	−	−	−	−	−	+	++	++	++	++
Cerebral Cortex	−	−	−	−	−	−	+	++	++	++
Hippocampus	−	−	−	−	−	−	+	++	++	++

Estimation of cholinergic neuron distribution patterning: −, not cholinergic; +, cholinergic but different from adult; ++, adult-like.

a The results of the serial section brain reconstructions have been revised according to the tdTomato enhancement stains.

b High cell density in caudal and ventral areas but low density in rostral and dorsal areas of the CPu.

c All brains with low intensity tdTomato-positive population in some but not all brain slices.

d This structure does not contain ACh neuron soma, assessment reflects innervation.

See also Extended Data Figures 5-1 and 5-2.

10.1523/ENEURO.0542-23.2024.t2-1 Table 2-1 Data table for mean fluorescence intensity for cortical layers at P3, P6, and P9 (refers to Fig 4 and Extended Data Fig 7). Download Table 2-1, DOC file.

Concomitantly, prenatal exposure to nicotine or pesticides that alter acetylcholinesterase activity, such as organophosphates or carbamates, are major risk factors for autism spectrum disorders (ASDs) or intellectual disability (Dwyer et al., 2008; Braun et al., 2009; Suarez-Lopez et al., 2013; Tran et al., 2013; Shelton et al., 2014; Browne et al., 2016; Guo et al., 2019; Semick et al., 2020). In fact, increased cholinergic signaling alleviates compulsive behaviors or symptoms in ASD in mice, while decreased cholinergic signaling facilitates the symptoms (Karvat and Kimchi, 2014; Martos et al., 2017; Mitra et al., 2016). Therefore, a better understanding of the development of and pathogenic interactions with the cholinergic system may offer potential treatment strategies for brain disorders, such as ASD or TS.

Despite recent breakthroughs, such as reconstruction of individual ACh neuron projection fields, the generation of whole-brain atlases of ACh neurons or input/output mapping to/from the basal forebrain ACh neurons, and RNA expression patterns in the developed brain, very little progress has been made regarding a comprehensive assessment of developmental changes in ACh neurons (Oh et al., 2014; Wu et al., 2014; Gielow and Zaborszky, 2017; Li et al., 2018; Cizeron, 2020; Wang et al., 2020; Tomas-Roca et al., 2022; Gamage et al., 2023). While there is a trove of literature about nAChR mRNA or protein level changes in many brain regions in the pre- or postnatal rodent brain, there is little data about how onset of cholinergic signaling overlaps with the nAChR expression patterns in these brain regions (Cimino, 1995; Jaarsma et al., 1997; Zhang, 1998; Abreu-Villaca et al., 2011; Zhang et al., 2016; Broide et al., 2019; Abbasi et al., 2020; Alzu’bi et al., 2020; Colangelo et al., 2022; Saad et al., 2022).

However, we need to assess the temporal changes in ACh neurons and cholinergic innervation during brain development to understand the role and disruption of cholinergic signaling in brain development. Therefore, we assessed how ACh neuron populations change in the developing pre- and postnatal brain. Of particular interest to us was during which time windows ACh neurons start to express the cholinergic genes, choline acetyltransferase (ChAT) and vesicular ACh transporter (vAChT, Cho et al., 2014). To identify ACh neuron populations during development, we reconstructed serial sectioned brains imaged with a confocal fluorescence microscope. We found three crucial time windows—two in the pre- and one in the postnatal brain—during which most of the ACh neuron populations become cholinergic. We observed that ACh neuron populations become cholinergic within a bit more than 1 week after birth in the mouse brain and before the onset of major sensory processes, such as vision and hearing. Furthermore, we detected a high synchrony of ACh neurons becoming cholinergic within individual nuclei as well as brain anatomical structures. Lastly, we assessed changes in cholinergic innervation in the striatum, cerebellum, cerebral cortex, and hippocampus. We found that cholinergic fibers innervate first the deep and superficial layers of the prefrontal and primary somatosensory cortex, before innervating the intermediate layers. The changes in innervation coincide with the activation of cholinergic gene expression in cortical and hippocampal ACh interneurons. Taken together, our data provides a comprehensive characterization when ACh neurons become cholinergic in the pre- and postnatal mouse brain that will be useful for basic research, understanding disease mechanisms, and neurotoxicology.

## Materials and Methods

### Animals

All animal procedures were approved by and performed in compliance with the NIEHS/NIH Humane Care and Use of Animals Protocols. All transgenic animal lines were purchased from The Jackson Laboratory and subsequently maintained and bred in-house. Animals were group housed (≤5 per cage) in a regular 12 h light/dark cycle under constant temperature control. Food and water were supplied *ad libitum*. We specifically labeled ACh neurons by crossing homozygous ChAT-IRES-Cre (Jax# 006410, RRID:IMSR_JAX:006410; Rossi et al., 2011) females with homozygous male Ai9 or Ai14 reporter mice (Jax# 007909 RRID:IMSR_JAX:007909, 007914 RRID:IMSR_JAX:007914; Madisen et al., 2010) on a C57BL/6J background. To collect embryonic brains, time mated females were checked daily for plugs, and the presence of plugs was considered embryonic day 0 (E0). Pregnant dams were monitored daily for new births, and the presence of newborn pups was considered postnatal day 0 (P0). The animals used in experiments were genotyped for transgenes and Y chromosome to verify sex (Extended Data Table 1).

### Histology

We collected brains of mice of either sex at E12, E15, and E18. To this end, pregnant dams were anesthetized with isoflurane and decapitated. Afterwards, embryos were removed, washed in ice-cold 0.1 M phosphate buffered saline (PBS), and fixed in 0.1 M PBS + 4% paraformaldehyde (PFA) at 4°C overnight. We collected postnatal brains at P1, P3, P6, P9, P12, P21, P50, and >1 year. Younger pups (<P6) were decapitated and their brains immersed in 0.1 M PBS + 4% PFA at 4°C overnight. Older mice (≥P6) were deeply anesthetized with sodium pentobarbital (100  mg/kg) and transcardially perfused with 0.1 M PBS and 0.1 M PBS + 4% PFA and postfixed in 0.1 M PBS + 2% PFA at 4°C overnight. Afterwards, the brains were freeze protected in 0.1 M PBS with 30% sucrose and frozen in Tissue-Tek O.C.T. Compound (Sakura Finetek).

For serial sectioning of entire brains, sagittal, horizontal, or coronal 50–100  µm slices were cut with a Leica CM3050 cryostat (Leica) and directly mounted on Superfrost Plus microscope slides (Thermo Fisher Scientific). After 15 min thawing, slides were washed in 0.01 M PBS for 5–10 min and air dried. Dry slices were stained with Hoechst 33342 using ProLong Glass Antifade Mountant with NucBlue Stain (Thermo Fisher Scientific) and protected with thickness 1.0 microscope cover glasses. Sectioned brains where more than two slices were lost during sectioning were discarded.

For immunostaining, horizontal 50 µm slices were cut with a cryostat and stored in Tris-buffered saline (TBS) + 50% glycerol at −20°C until use. Slices, ranging from Plate 137 to 160 according to the Paxinos Mouse Brain Atlas, containing the dorsal striatum, hippocampus, basal forebrain, cerebellum, and hindbrain, were chosen for immunostaining (Keith and Franklin, 2013). Slices were rinsed three times in TBS. After washing, slices were blocked in TBS + 0.3% Triton X-100 (Thermo Fisher Scientific) + 10% normal donkey serum (NDS, Abcam) or normal goat serum (NGS, Abcam), depending on secondary antibody donor species, at RT for 2 h. Afterward, slices were incubated with primary antibodies (see Table 1) diluted in TBS + 0.3% Triton X-100 + 5% NDS/NGS at 4°C overnight. After three times washing in TBS, slices were incubated with secondary antibodies (see Table 1) diluted in TBS + 0.3% Triton X-100 + 5% NDS/NGS at 4°C overnight. Slices were washed three times in TBS before they were mounted on Superfrost Plus microscope slides, air dried, and protected with ProLong Glass Antifade Mountant with NucBlue (Thermo Fisher Scientific) and microscope cover glasses. To ensure antibody staining specificity, every round of staining included several slices which were unstained or only treated with secondary antibody.

### Fluorescence confocal microscopy

For serial brain section imaging, fluorescent images were collected with an inverted LSM 710 laser scanning confocal microscope (Zeiss). Fluorophores were excited with a 405 nm diode (Hoechst 33342) and a 561 nm DPSS laser (tdTomato). Multiple beam splitters MBS 488/561 and MBS −405 were used to separate fluorescent signals. We used a Plan-Apochromat 10x/0.45 M27 objective to collect single tile scans of individual brain slices with 0.83 µm × 0.83 µm pixel size, 16 bit depth, and 10% tile overlap. The pinhole was adjusted to 1 AU for the longest wavelength fluorophore and fluorescence intensity to below saturation levels. Microscope settings were maintained throughout the experiment for all imaged brains for the tdTomato signal but with minor adjustments (<20%) in gain for the Hoechst 33342 channel.

For immunohistochemistry, fluorescent images were collected with an inverted LSM 980 laser scanning confocal microscope (Zeiss) controlled by the software Zen blue (Zeiss). Fluorophores were excited with a 405 nm diode (Hoechst), a 561 nm DPSS laser (tdTomato), and a 639 nm diode laser (A647). Multiple beam splitters MBS 488/561/639 and MBS −405 were used to separate fluorescent signals. The software semiautomatically detected brain slices on a slide and adjusted focus. Tile scans of individual brain slices were taken at 16 bit depth using either (1) AC-Plan-Neofluar 10x/0.3 M27 objective with 0.83 µm × 0.83 µm pixel size to image ChAT/tdTomato or NeuN stains or (2) Plan-Apochromat 20x/0.8 M27 objective with 0.41 µm × 0.41 µm pixel size to image cholinergic fibers after tdTomato enhancement stains.

For ChAT/tdTomato colocalization experiments, individual brain regions of interest containing tdTomato-positive cells were imaged as *Z*-stacks. Images were acquired using serial line scan. Z-stacks were scanned through the whole slice *z* range (typically ∼60 µm thickness, 4 µm *z*-step). The pinhole was always adjusted to 1 AU for the longest wavelength fluorophore and fluorescence intensity to below saturation levels. Microscope settings were maintained throughout the experiment for all images for the fluorescent signals without changes in gain or laser intensity, including the control or unstained slices where applicable.

### Image processing and brain registration of serial sectioned brains

Tile scans of individual brain slices were stitched using Zen Black (Zeiss, RRID:SCR_018163) using a correlation threshold >0.9. Stitched brain slices were background corrected in ImageJ using a rolling ball 50 pixels value and semiautomatically aligned using BrainMaker (RRID:SCR_017346, MBF Bioscience). Stacks of brain slices were imported into Imaris 9.7.2 or higher (RRID:SCR_007370, Bitplane) for three-dimensional presentation and cell identification. Three reference mouse brain atlases were used for brain region identification (Paxinos et al., 2020; Keith and Franklin, 2013; Schambra, 2008). Brain regions containing tdTomato-positive cells were identified visually and manually outlined as well as depicted using the “Surfaces” function in Imaris. Movies were rendered using the “Animation” option in Imaris to visualize individual brain reconstructions.

#### Quantitative image analysis

To assess the labeling efficiency of the ChAT-IRES-Cre × Ai14 animal model for cholinergic neurons by brain region, individual tdTomato or ChAT positive cells were counted manually in *z*-stacks from individual brain regions using ImageJ (RRID:SCR_003070, www.imagej.net). Ratios of tdTomato/ChAT co-positive cells were calculated per *z*-stack and averaged by brain region.

To quantify the cholinergic innervation of the primary somatosensory cortex, the ImageJ tools “Polygon” and “Measure” were used to manually outline individual cortical layers identified by the Hoechst 33342 signal and then extract mean fluorescence intensity values for the A647 channel. Only the layers for one area per hemisphere per slice were assessed. Only areas without distortions, artifacts, or cholinergic interneurons were used for quantification. The analysis was not performed for the ages P12 and P50 due to the large number and potentially biasing effects on fluorescence intensity of local cholinergic interneurons. To ensure a reliable identification of the layers at P3-P9, L2/3, and L4 were combined. For comparison, fluorescence values of control slices without primary and/or secondary antibody were quantified in the same way. To increase the number of control values, areas of the insular, secondary somatosensory, and ventral auditory cortices were added. The standard deviation of the mean fluorescence intensity for control slices was consistently below 10% of the mean for all layers and ages.

### Statistics

The experiments were performed with at least three biological replicates per condition. For immunostainings, brain regions were assessed in several slices per animal to ensure reproducibility. Average fluorescence intensities for individual cortical layers were compared with each other and control slices not treated with primary and/or secondary antibody. A Brown–Forsythe and Welch analysis of variance (ANOVA) with correction for multiple comparisons by controlling the false discovery rate with the Benjamini, Krieger, and Yekutieli method was performed using Prism 10 (RRID:SCR_002798, GraphPad Software). Statistically significant differences were assumed with values of *q* < 0.05 and *p* < 0.05. An ordinary one-way ANOVA with Bonferroni’s post hoc correction for multiple comparisons yielded comparable results. Data is presented as mean ± standard deviation (σ). Box plots represent 10–90 percentile distributions.

## Results

### ACh neuron populations are efficiently labeled in the mature mouse brain in transgenic ChAT-IRES-Cre crossed with tdTomato reporter mice

Our goal was to assess when ACh neuron populations become cholinergic in the mouse brain during development. We define “becoming cholinergic” as the initiation of cholinergic gene expression from the combined *Chat/Slc18a3* locus to allow the neuron for the synthesis of ACh and transport into synaptic vesicles (Erickson et al., 1994). For this purpose, we selectively labeled ACh neurons with genetically encoded tdTomato by crossing the transgenic ChAT-IRES-Cre driver with Cre tdTomato reporter mouse lines. The presence of tdTomato would indicate which ACh neuron populations have a cholinergic fate.

First, we verified a high labeling efficiency for the animal model by comparing the overlap of tdTomato-positive cells with the ACh neuron marker ChAT throughout the mature mouse brain (Extended Data Fig. 1-1). All major brain regions with ACh neurons had at least 70% ChAT-positive cells colabeled with tdTomato (Extended Data Fig. 1-1*I*). In addition, the tdTomato signal was strong in cells with low ChAT expression. This means that this animal model can be used to efficiently label ACh neurons in the mouse brain.

### Many ACh neuron populations are cholinergic in the prenatal brain

**Figure 1.**
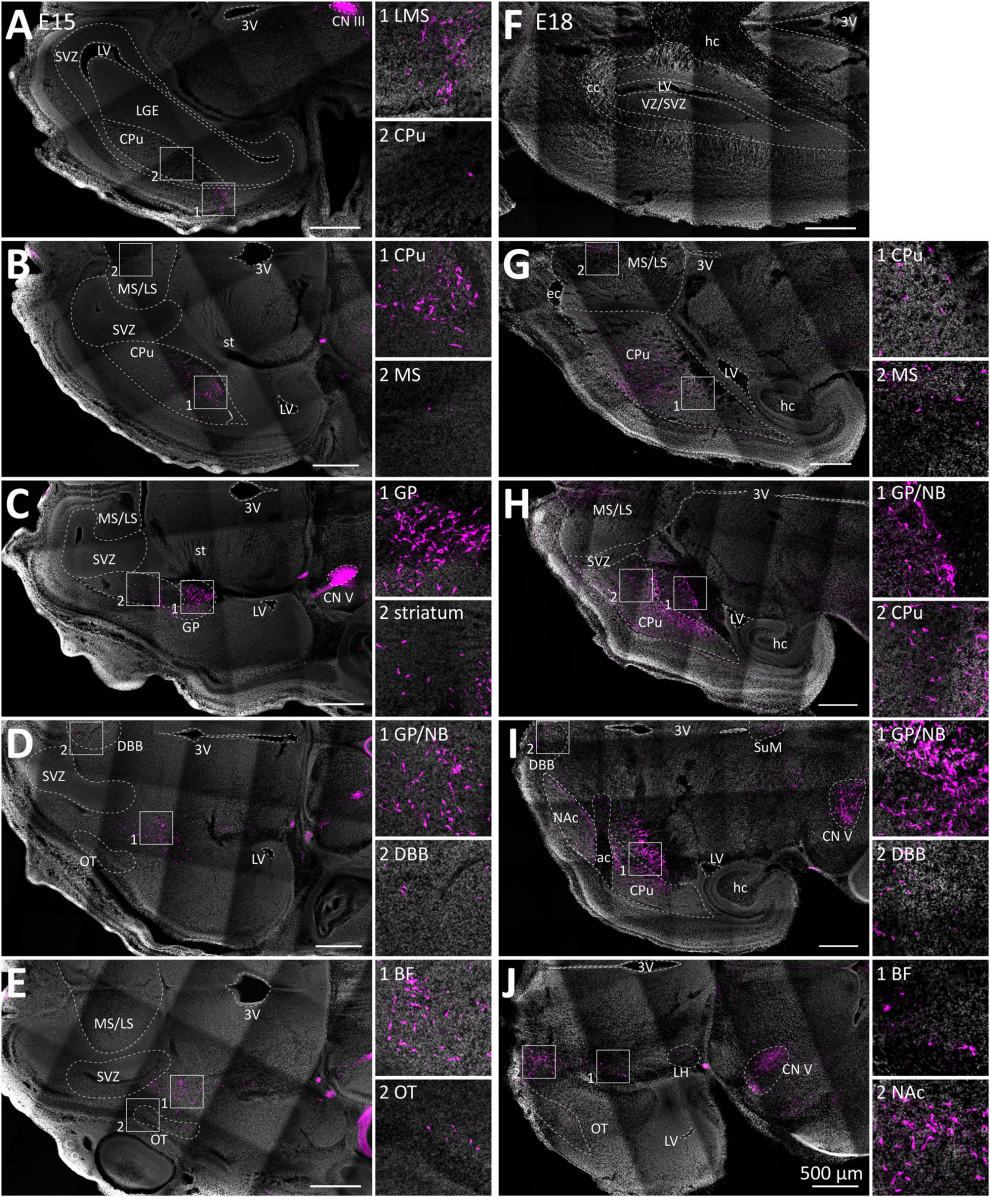
Distribution of ACh neurons in the mouse brain at E15 and E18. 50 µm horizontal brain sections of mice expressing tdTomato in ACh neurons at E15 and E18 were stained for DsRed. Sections were stained for DsRed to enhance tdTomato signal (magenta) and Hoechst 33342 (white). Brain regions were identified according to horizontal brain atlases of the prenatal mouse brain (Schambra, 2008). ***A***, This dorsal E15 slice contains ventricular and subventricular zone (SVZ), lateral ganglionic eminence (LGE, striatal neuroepithelium), lateral ventricle (LV), caudate putamen (CPu), third ventricle (3 V), and cranial nerve 3 (CN III). Insets show ACh neurons within the CPU, and the lateral migratory stream (LMS), depicting potentially migratory ACh neurons. ***B***, A more ventral slice contains medial septal and lateral septal nuclei (MS/LS), stria terminalis (st), 3 V, and CPu. As shown in the insets, ACh neurons were detected in the caudal CPu and MS. ***C***, A more ventral slice contains ACh neurons in the globus pallidus (GP), striatum, and cranial nerve 5 (CN V). ***D***, A more ventral slice contains ACh neurons in the GP/nucleus basalis of Meynert (NB), the vertical limb of the diagonal band of Broca (DBB), and the olfactory tubercle (OT). ***E***, The most ventral slice contains ACh neurons in OT and basal forebrain nuclei (BF). ***F***, A dorsal E18 slice contains brain regions VZ/SVZ, LV, corpus callosum (CC), and hippocampus (hc). ***G***, More ventral, ACh neurons are cholinergic in the CPU and MS/LS. ***H***, A more ventral slice contains cholinergic neurons in the MS/LS, GP/NB, and CPu. ***I***, In a more ventral slice, additional cholinergic neurons are located in the DBB, nucleus accumbens (NAc), supramammillary nucleus (SuM), and CN V. The anterior commissure (ac) separates ventral and dorsal striatum. ***J***, The most ventral slice contains additional cholinergic neurons in BF, OT, and the lateral hypothalamus (LH). See Extended Data Figures 1-2 and 1-3 for more details.

To assess ACh neuron populations in the prenatal brain, we fixed brains at E15 and E18. We stained brain slices for DsRed to enhance the signal for tdTomato. Individual brain regions were identified by comparing both the Hoechst 33342 and autofluorescence signals with different brain atlases. In the prenatal brain at E15, we detected ACh neurons in cranial nerve (CN) nuclei of the midbrain, pons, and medulla by their intrinsic tdTomato fluorescence. In the forebrain, only the enhanced tdTomato signal allowed for the reliable detection of ACh neurons in the striatum and the basal forebrain (Fig. 1). One group of cholinergic neurons was detected in the lateral migratory stream indicating that some ACh neurons may be cholinergic before reaching their destination (Fig. 1*A*). In the caudate putamen (CPu) at E15, ACh neurons are cholinergic particularly on the caudal and ventral side (Fig. 1*A*,*B*). More ventral in the brain, ACh neurons are densely clustered in the globus pallidus (GP, Fig. 1*C*). In the basal forebrain, ACh neurons are already cholinergic in basal forebrain nuclei, such as the vertical limb of the diagonal band of Broca (DBB), the ventral pallidum (VP), the nucleus basalis of Meynert (NB), and the substantia innominata (SI, Fig. 1*D*,*E*). Further tdTomato-positive cells were detected in the ventral striatum both in nucleus accumbens and olfactory tubercle (Fig. 1*D*,*E*). However, proliferation zones, such as the lateral ganglionic eminence or the ventricular/subventricular zone, are mostly free of tdTomato-positive cells with only a few cells in some caudal areas. At E18, however, cholinergic neurons in the basal ganglia and the basal forebrain are generally better organized (Fig. 1*G–J*). In addition, some hypothalamic nuclei, such as the supramammillary nucleus (SuM) and the lateral hypothalamus (LH) contain tdTomato-positive cells indicating the onset of cholinergic gene expression (Fig. 1*I*,*J*).

10.1523/ENEURO.0542-23.2024.f1-1 Fig 1-1 **ACh neurons are reliably labeled throughout the brain using the ChAT-IRES-Cre x Ai14 tdTomato mouse model.** A-I, 50 µm horizontal brain sections of mice expressing tdTomato in ACh neurons were obtained at P50 and immunostained for ChAT. Sections were imaged for tdTomato (green) and ChAT (magenta) signals using a confocal laser scanning fluorescence microscope. Maximum intensity projections of ∼50 µm z stacks of brain regions imaged with the confocal microscope include: caudate putamen (CPu, A), laterodorsal tegmentum and pedunculopontine nucleus (LDT/PPN, B), cranial nerve V (CN V, C), cranial nerve X and XII (CN X/XII, D), cerebral cortex (E), lateral septal complex (LS, F), medial septum and diagonal band of Broca (MS/DBB, G), and nucleus basalis of Meynert and globus pallidus external (NB/GP, H). Insets depict magnified ROIs outlined by the small white square. I, Mean ratio ± standard deviation of tdTomato- and ChAT-positive cell overlap per brain region. Numbers inside the bar graphs indicate individual z-stacks counted. Download Fig 1-1, TIF file.

**Figure 2.**
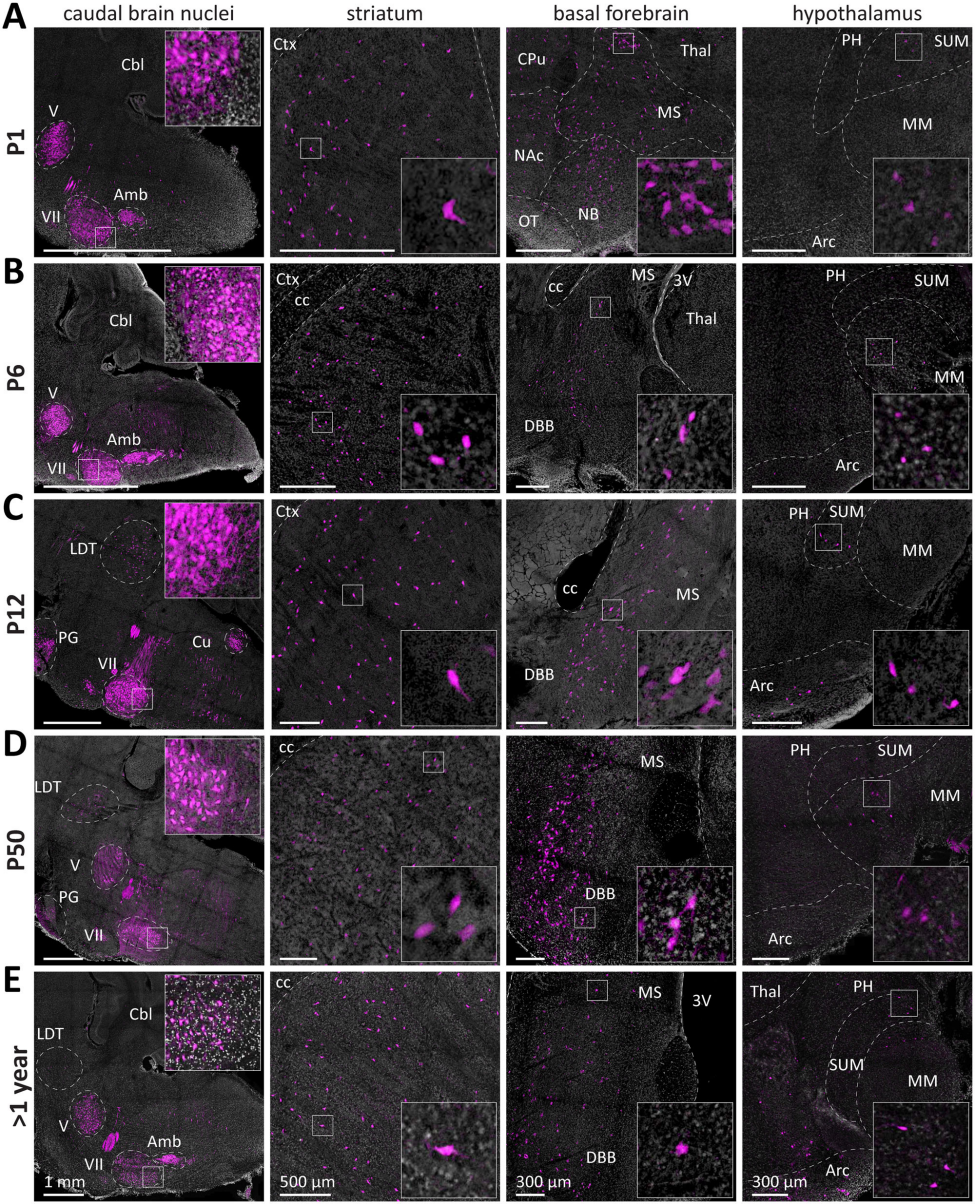
Several ACh neuron populations are cholinergic in the neonatal mouse brain. ***A–E***, 100 µm sagittal brain sections of mice expressing tdTomato in ACh neurons were obtained at P1 (***A***), P6 (***B***), P12 (***C***), P50 (***D***), and 1 year of age (***E***). Sections were imaged for tdTomato (magenta) and DAPI (white) signals using a confocal laser scanning fluorescence microscope. These brain regions were identified: caudal brain nuclei in the brainstem and midbrain (left), striatum (second left), basal forebrain (second right), hypothalamus (right). Insets depict magnified ROIs outlined by the small white square. At P1, ACh neurons are present in the striatum, basal forebrain, hypothalamus, and caudal brain nuclei. Abbreviations: Ctx, cerebral cortex; cc, corpus callosum; NAc, nucleus accumbens; OT, olfactory tubercle; DBB, diagonal band of Broca; MS, medial septum; 3V, third ventricle; cbl, cerebellum; V, CN V; VII, CN VII; Amb, nucleus ambiguus; Cu, cuneate nucleus; PG, pontine gray; LDT, laterodorsal tegmentum; Thal, thalamus; PH, posterior hypothalamic nucleus; SUM, supramammillary nucleus; MM, medial mammillary nucleus.

10.1523/ENEURO.0542-23.2024.f1-2 Fig 1-2 **Distribution of ACh neurons compared to neuron marker NeuN in the mouse brain at E15.** A-H, 50 µm horizontal brain sections of mice expressing tdTomato in ACh neurons at E15 were immunostained for NeuN. Sections were imaged for tdTomato (green) and NeuN (magenta). Brain regions imaged include: CPu (A), LDT/PPN (B), CN V (C), cranial nerve III/IV (CN III/IV, D), NB/GP (E), cortical plate (F), cerebellum (cbl, G), and hippocampus (hc, H). Insets depict magnified ROIs outlined by the small white square. Download Fig 1-2, TIF file.

**Figure 3.**
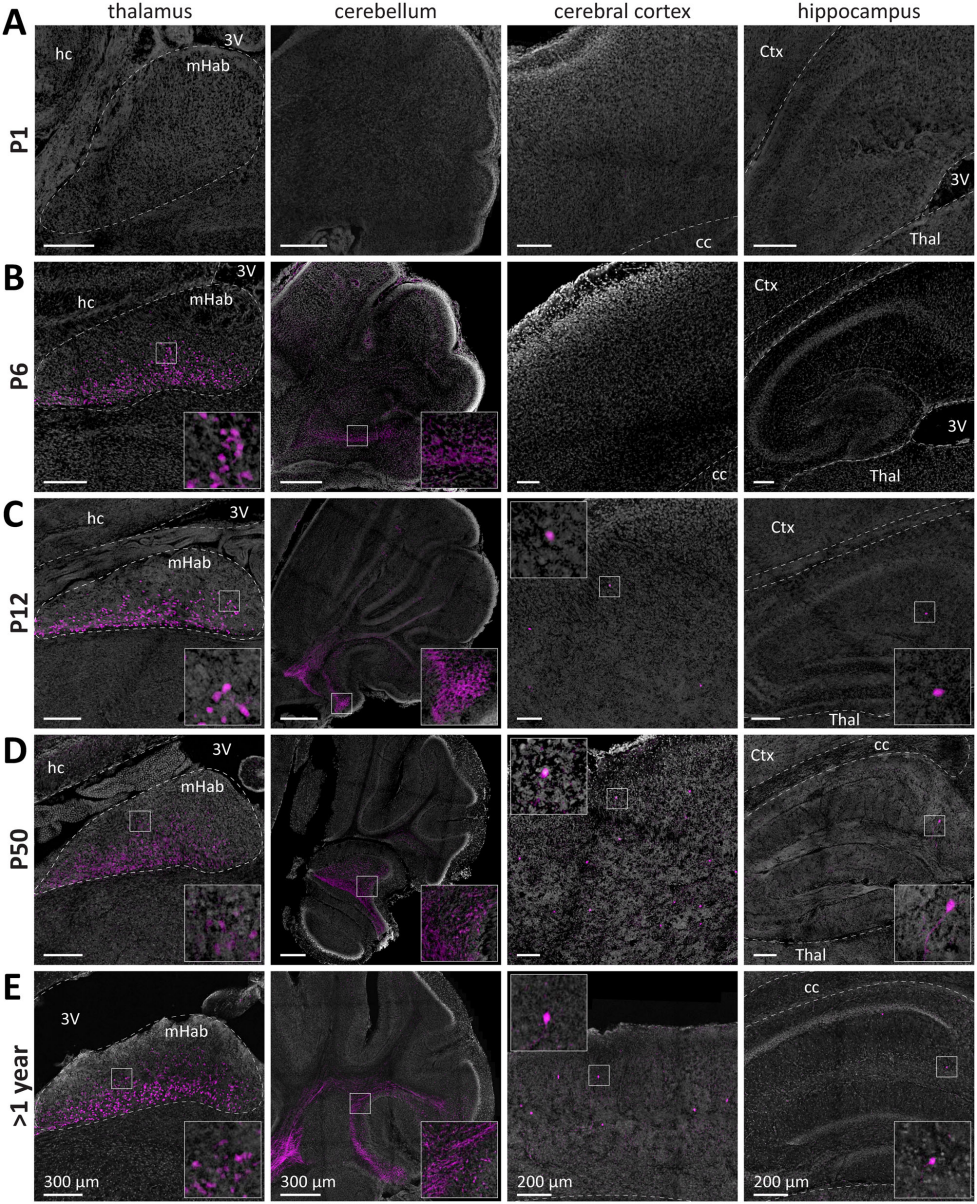
Several ACh neuron populations are not cholinergic in the neonatal mouse brain but are found cholinergic at later ages. ***A–E***, Brain sections of the mice from Fig. 1 were assessed for tdTomato fluorescence at P1 (***A***), P6 (***B***), P12 (***C***), P50 (***D***), and 1 year (***E***) in these brain regions: thalamus (left), cerebellum (second left), cerebral cortex (second right), and hippocampus (right). Insets depict magnified ROIs outlined by the small white square. At P1, ACh neurons are absent in the thalamus, cerebellum, cerebral cortex, and hippocampus. At P6, ACh neurons are present in the thalamus and cerebellum. At P12, P50, and 1 year, ACh neurons were present in all brain regions. Abbreviations: Ctx, cerebral cortex; cc, corpus callosum; 3V, third ventricle; hc, hippocampus; Thal, thalamus; mHab, medial habenula.

10.1523/ENEURO.0542-23.2024.f1-3 Fig 1-3 **Distribution of ACh neurons compared to neuron marker NeuN in the mouse brain at E18.** A-H, 50 µm horizontal brain sections of mice expressing tdTomato in ACh neurons at E18 were immunostained for NeuN. Sections were imaged for tdTomato (green) and NeuN (magenta). Brain regions imaged include: CPu (A), LDT/PPN (B), CN V (C), CN III/IV (D), NB/GP (E), cortical plate (F), cerebellum (G), and hippocampus (H). Insets depict magnified ROIs outlined by the small white square. Download Fig 1-3, TIF file.

For comparison, we performed counterstains for NeuN to assess the patterning of postmitotic neuron populations in the brain (Extended Data Fig. 1-2; Mullen et al., 1992). In general, the NeuN patterning changes little from E15 to E18 except for the cerebellum, hippocampus, and cortical plate (Extended Data Figs. 1-2, 1-3*F–H*). In the hindbrain, the tdTomato signal overlaps well with NeuN only in the nucleus of the trigeminal nerve (CN V; Extended Data Figs. 1-2, 1-3*C*). Taken together, this data indicates that ACh neurons in the forebrain become cholinergic at or before E15 and are well organized on a soma level by E18.

### Many ACh neuron populations are cholinergic in the neonatal mouse brain

In the neonatal brain (P1), we detected ACh neurons in nuclei of the midbrain, pons, medulla, striatum, hypothalamus, and basal forebrain (Fig. 2). For our assessment, we combined the regions of the midbrain, pons, and medulla as caudal brain nuclei. In those regions reside both cranial nerves (CN) and other nuclei. While ACh neurons in many caudal brain nuclei are densely packed, such as in the CNs, the cuneate nucleus (Cu), or the pontine gray (PG), ACh neurons in nuclei, such as the pedunculopontine nucleus (PPN) or laterodorsal tegmentum (LDT), are sparse. Furthermore, there are substantial differences in soma diameter between the individual nuclei ranging from 10 to >20 µm. We detected various thick bundles of processes projecting within the brain region, to other brain regions, or targets outside of the brain mostly along the dorsoventral axis. In the striatum, we detected a sparse population of mostly multipolar neurons with a large soma diameter (∼18 µm). In the basal forebrain, we observed a heterogeneous neuron distribution pattern, soma size, and cell complexity. Occasionally, septohippocampal ACh projections are visible for individual neurons in the most dorsal parts of the medial septum. In the hypothalamus, we first detected sparse cell populations mostly in areas around the midline in posterior areas, such as the supramammillary nucleus. At later ages, ACh neurons are also present in more anterior, ventral areas such as the arcuate nucleus. However, the overall number of tdT-positive cells in the hypothalamus is low. Overall, in the mature brain at P50 and 1 year of age, the ACh neuron populations do not appear different from the developing brain.

### Some ACh neuron populations are not cholinergic in the neonatal mouse brain

In the neonatal brain (P1), we did not detect ACh neurons or cholinergic innervation in the thalamus, cerebellum, cerebral cortex, and hippocampus (Fig. 3). At P6, however, additional tdTomato-positive neurons or innervation were detected in the thalamus and fibers in the cerebellum (Fig. 3*A*,*B*). In the thalamus, we observed a heterogeneous ACh neuron distribution. Most neurons are densely packed in the medial habenula (mHab), with a slight ventrodorsal gradient. In general, thalamic ACh neurons have small soma diameters (∼9 µm). The fiber bundles from the mHab to the midbrain are also evident as early as P6.

In the cerebellum, the first parts innervated by ACh neurons are the paraflocculus as well as posterior parts of the lateral cerebellum adjacent to it. From there, the tdTomato signal spreads medial to the posterior parts of the cerebellar vermis as well as the anterodorsal parts of the lateral cerebellum. At later time points, the tdTomato signal shows a pronounced distribution gradient, with lateral and ventral parts showing the highest, but medial and dorsal parts the lowest coverage. Cholinergic fiber coverage is predominantly found in the white matter, some in the granular and Purkinje layers but none in the molecular layer.

At P12, both the hippocampus and cerebral cortex contain sparse ACh neuron populations (Fig. 3*C*). In the cerebral cortex, there are mostly bipolar cells with small soma diameters (∼11 µm) predominantly in L2/3 and some in L5. In the hippocampus, we detected the sparsest ACh neuron population in the brain composed of both bipolar and multipolar neurons (∼30–50 neurons per hemisphere at P50). ACh neuron soma are of intermediate size (∼14 µm diameter) and about equally distributed across all hippocampal areas with a slight preference for the dorsal subiculum.

While we detected considerably increased ACh innervation particularly in the cortex at later ages, no further populations were detected in the mature brain at P50 and 1 year of age (Fig. 3*D*,*E*). We, therefore, conclude that ACh neuron soma are present and cholinergic in all brain regions at or before P12.

### Cholinergic innervation of forebrain regions is gradually refined during development

**Figure 4.**
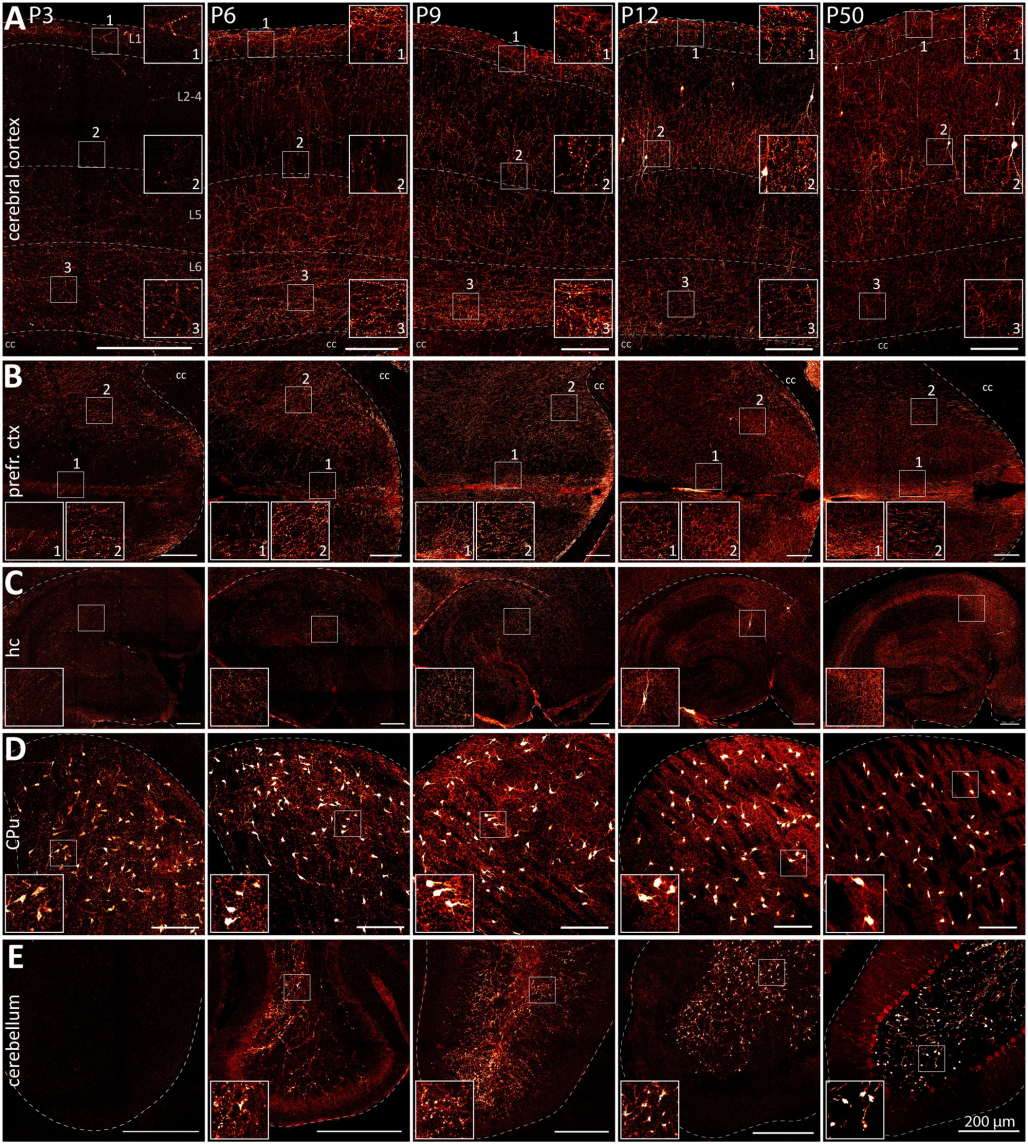
Cholinergic innervation of major brain regions changes substantially in the postnatal mouse brain between P3 and P50. ***A–E***, Horizontal 50 µm brain sections were stained for DsRed to enhance tdTomato signal and better visualize cholinergic projections in the cerebral cortex (primary somatosensory cortex S1, ***A***), prefrontal cortex (prefr. ctx, area 24, area 32, medial orbitofrontal cortex MO, ***B***), hippocampus (***C***), CPu (***D***), and cerebellum (***E***). ***A***, ***B***, At P3, cholinergic fibers are dense in L6, sparser in L1, and sparsest in intermediate L2–5. At P6, cholinergic fibers are dense in deep and superficial cortical layers but sparse in intermediate cortical layers. At P9–P12, intermediate layers become more densely innervated but there remain gaps in innervation between individual layers. At P50, gaps in cholinergic innervation are closed. Borders of cortical layers L1, L2–4, L5, and L6 are indicated by dotted lines. ***C***, Hippocampal cholinergic innervation is sparse at P3–P9 compared with P12–P50. ***D***, In the dorsal striatum, there are areas which are avoided by cholinergic fibers. This compartmentalization becomes more pronounced from P12 to P50. ***E***, In the cerebellum, cholinergic innervation is absent at P3 and at P6 sparse with little organization. Throughout development, fiber bundles and cholinergic terminals become better organized as well as structurally complex. Color coding corresponds to fluorescence intensity with red for low and white for high intensity, respectively. Insets depict magnified ROIs outlined by the small white square. Abbreviations: cc, corpus callosum. See Extended Data Figures 4-1–4-4 for more details.

In addition to the changes in ACh neuron soma distribution, we assessed changes in cholinergic innervation during development. For this purpose, we amplified the tdTomato signal by immunostaining and imaged stained brain slices (Fig. 4). We further compared our findings on cholinergic innervation with stainings for NeuN at P3, P6, and P12 to get a better overview about the distribution of mature neurons in the brain (Extended Data Figs. 4-1–4-3).

10.1523/ENEURO.0542-23.2024.f4-1 Fig 4-1 **Distribution of ACh neurons compared to neuron marker NeuN in the mouse brain at P3.** A-H, Brain sections obtained at P3 were assessed as described in Figure 1-2. Imaged brain regions include: CPu (A), LDT/PPN (B), CN V (C), CN III/IV (D), NB/GP (E), S2/AuV (F), cerebellum (G), and hippocampus (H). Insets depict magnified ROIs outlined by the small white square. Download Fig 4-1, TIF file.

10.1523/ENEURO.0542-23.2024.f4-2 Fig 4-2 **Distribution of ACh neurons compared to neuron marker NeuN in the mouse brain at P6.** A-H, Brain sections obtained at P6 were assessed as described in Figure 1-2. Imaged brain regions include: CPu (A), LDT/PPN (B), CN V (C), CN III/IV (D), NB/GP (E), S2/AuV (F), cerebellum (G), and hippocampus (H). Insets depict magnified ROIs outlined by the small white square. Download Fig 4-2, TIF file.

10.1523/ENEURO.0542-23.2024.f4-3 Fig 4-3 **Distribution of ACh neurons compared to neuron marker NeuN in the mouse brain at P12.** A-H, Brain sections obtained at P12 were assessed as described in Figure 1-2. Imaged brain regions include: CPu (A), LDT/PPN (B), CN V (C), CN III/IV (D), NB/GP (E), S2/AuV (F), cerebellum (G), and hippocampus (H). Insets depict magnified ROIs outlined by the small white square. Download Fig 4-3, TIF file.

10.1523/ENEURO.0542-23.2024.f4-4 Fig 4-4 **Quantification of cholinergic innervation of S1 layers at P3, P6, and P9.** A-C, Box plots depicting the average fluorescence intensity as a measure for cholinergic innervation. Cholinergic innervation of S1 L1 (orange) and L6 (blue) is significantly higher than L2-4 (yellow) and L5 (green) from P3-P9. L2-4 and L5 are not significantly different from each other from P3-P9. A Brown-Forsythe and Welch ANOVA with correction for multiple comparisons by controlling the false discovery rate with the Benjamini, Krieger, and Yekutieli method was used to assess differences. Only statistically significant comparisons (q<0.05, p<0.05) are shown. Download Fig 4-4, TIF file.

At P3, the deep part of L6 and L1 in the barrel field of the primary somatosensory cortex (S1) are densely innervated, while the other layers remain sparsely innervated with cholinergic fibers (Fig. 4*A*, left; Extended Data Fig. 4-4; Extended Data Table 2). Likewise at P6, the deep and superficial layers in S1 are densely innervated, while intermediate layers L2–5 remain sparsely innervated with cholinergic fibers predominantly passing through (Fig. 4*A*, second left; Extended Data Fig. 4-4*B*; Extended Data Table 2). At P9 and P12, L2–5 become more densely innervated, but there is a clear separation of cholinergic innervation by layer and a markedly decreased fluorescence intensity compared with L1 and L6 (Fig 4*A*, second right; Extended Data Fig 4-4*C*; Extended Data Table 2). At P50, cholinergic innervation is more homogenous without any gaps between the individual layers (Fig. 4*A*, right). The distribution of mature neurons in the cortex, however, does not change much between P6 and P12 according to the NeuN staining (Extended Data Figs. 4-1–4-3).

Similarly, the prefrontal cortex areas (areas 24, 32 of the anterior cingulate cortex, as well as medial orbitofrontal cortex, MO) already contain dense cholinergic innervation in deep layers (Fig. 4*B*, left). The innervation of superficial layers can be detected at P6 with a pronounced innervation gap in intermediate layers (Fig. 4*B*, second left). At later time points, the gap in cholinergic innervation of intermediate layers is closed (Fig. 4*B*, center, second right, right). Furthermore, the cholinergic innervation in superficial layers becomes denser between P12 and P50.

In the hippocampus at P3 and P6, cholinergic innervation shows little organization and mostly innervates the molecular layer of the dentate gyrus (Fig. 4*C*, left, second left). While hippocampal neurons appear well organized as early as P6 according to the NeuN stain, only cholinergic innervation at P12 and P50 appears compartmentalized by hippocampal subregion (Fig. 4*C*, second right, right; Extended Data Fig. 4-2*H*).

In the CPu at P3 and P6, cholinergic innervation shows slight compartmentalization with small spots of decreased cholinergic fiber density (Fig. 4*D*, left, second left). The NeuN staining, however, indicates strong neuron compartmentalization as early as P3 (Extended Data Fig. 4-1). At later time points, compartmentalization of cholinergic fibers becomes more pronounced (Fig. 4*D*, center, second right, right).

At P3, cerebellar cholinergic innervation is absent (Fig. 4*E*, left). At P6, cerebellar cholinergic innervation is sparse and not well structured (Fig. 4*E*, second left). At later time points, the distribution of cholinergic fibers becomes more consistent (Fig. 4*E*, center, second right, right). Furthermore, terminals exhibit pronounced synaptic swellings as early as P12. The NeuN staining indicates that cerebellar neurons are well organized as early as P6 (Extended Data Fig. 4-2).

Taken together, this data indicates that various brain regions are mostly innervated by approximately P12. However, the cholinergic innervation is further refined until at least P50 resulting in a stronger compartmentalization or refinement of cholinergic fibers in the mature brain.

### A pre- and postnatal timeline when ACh neuron populations become cholinergic in the mouse brain

**Figure 5.**
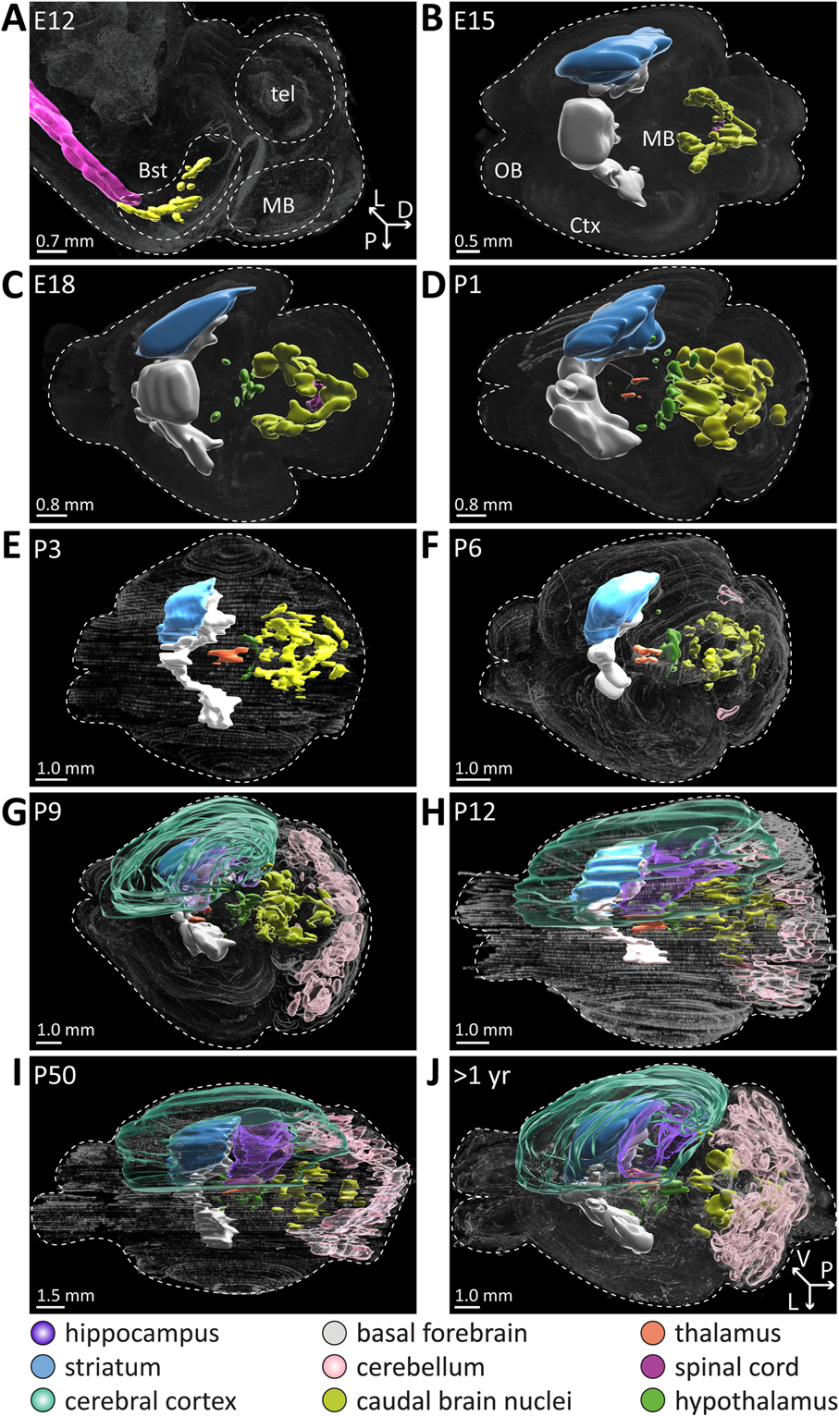
ACh neuron populations become cholinergic at different times during development. Reconstructions of serial sectioned brains were collected at E12 (***A***), E15 (***B***), E18 (***C***), P1 (***D***), P3 (***E***), P6 (***F***), P9 (***G***), P12 (***H***), P50 (***I***), and >1 year of age (***J***). For better visibility, diagrams of ACh populations were added color-coded by brain regions: transparent dark purple, hippocampus; light blue, striatum; transparent light green, cerebral cortex; orange, thalamus; light gray, basal forebrain; transparent light pink, cerebellum; yellow, caudal brain nuclei; light purple, spinal cord; green, hypothalamus. Abbreviations: tel, telencephalon; MB, midbrain; OB, olfactory bulb. For clarity, the striatum, cerebral cortex, and hippocampus were outlined in only one hemisphere per brain. All brains viewed from the top except for E12 (side view). ACh neuron populations in the forebrain at E15 and E18 were added according to findings from tdTomato enhancement staining. See Movies 1–10 for rendered view from all sides. See Extended Data Figures 5-1 and 5-2 for more details.

Studying single slices precludes the thorough comparison and visualization of disparate samples, such as brains at different ages. Therefore, we refined our assessment when ACh neurons become cholinergic by employing reconstruction of the serial sectioned brains and extended the timeline to 10 different time points during pre- and postnatal development, from E12 to >1 year of age refined by additional findings using tdTomato enhancement staining results (Fig. 5, Movies 1–10).

10.1523/ENEURO.0542-23.2024.f5-1 Fig 5-1 **All reconstructed mouse brains from E12 to P3.** A-E, Reconstructions of serial sectioned brains were collected at E12 (A), E15 (B), E18 (C), P1 (D), P3 (E). For better visibility cartoons of ACh populations were added color-coded by brain regions: transparent dark purple – hippocampus, light blue – striatum, transparent light green – cerebral cortex, orange – thalamus, light gray – basal forebrain, light pink – cerebellum, yellow – caudal brain nuclei, light purple – spinal cord, green - hypothalamus. Download Fig 5-1, TIF file.

10.1523/ENEURO.0542-23.2024.f5-2 Fig 5-2 **All reconstructed mouse brains from P6 to >1 yr.** A-E, Reconstructions of serial sectioned brains were collected at P6 (A), P9 (B), P12 (C), P50 (D), and >1 year of age (E). For better visibility cartoons of ACh populations were added color-coded by brain regions: transparent dark purple – hippocampus, light blue – striatum, transparent light green – cerebral cortex, orange – thalamus, light gray – basal forebrain, transparent light pink – cerebellum, yellow – caudal brain nuclei, light purple – spinal cord, green - hypothalamus. Download Fig 5-2, TIF file.

10.1523/ENEURO.0542-23.2024.v1 Movie 1 Animated View at the cholinergic ACh neuron populations in E12 brain reconstruction depicted in Fig 5 A. Download Movie 1, MP4 file.

10.1523/ENEURO.0542-23.2024.v2 Movie 2 Animated View at the cholinergic ACh neuron populations in E15 brain reconstruction depicted in Fig 5 B. Download Movie 2, MP4 file.

10.1523/ENEURO.0542-23.2024.v3 Movie 3 Animated View at the cholinergic ACh neuron populations in E18 brain reconstruction depicted in Fig 5 C. Download Movie 3, MP4 file.

10.1523/ENEURO.0542-23.2024.v4 Movie 4 Animated View at the cholinergic ACh neuron populations in P1 brain reconstruction depicted in Fig 5 D. Download Movie 4, MP4 file.

10.1523/ENEURO.0542-23.2024.v5 Movie 5 Animated View at the cholinergic ACh neuron populations in P3 brain reconstruction depicted in Fig 5 E. Download Movie 5, MP4 file.

10.1523/ENEURO.0542-23.2024.v6 Movie 6 Animated View at the cholinergic ACh neuron populations in P6 brain reconstruction depicted in Fig 5 F. Download Movie 6, MP4 file.

10.1523/ENEURO.0542-23.2024.v7 Movie 7 Animated View at the cholinergic ACh neuron populations in P9 brain reconstruction depicted in Fig 5 G. Download Movie 7, MP4 file.

10.1523/ENEURO.0542-23.2024.v8 Movie 8 Animated View at the cholinergic ACh neuron populations in P12 brain reconstruction depicted in Fig 5 H. Download Movie 8, MP4 file.

10.1523/ENEURO.0542-23.2024.v9 Movie 9 Animated View at the cholinergic ACh neuron populations in P50 brain reconstruction depicted in Fig 5 I. Download Movie 9, MP4 file.

10.1523/ENEURO.0542-23.2024.v10 Movie 10 Animated View at the cholinergic ACh neuron populations in >1 year brain reconstruction depicted in Fig 5 J. Download Movie 10, MP4 file.

**Figure 6.**
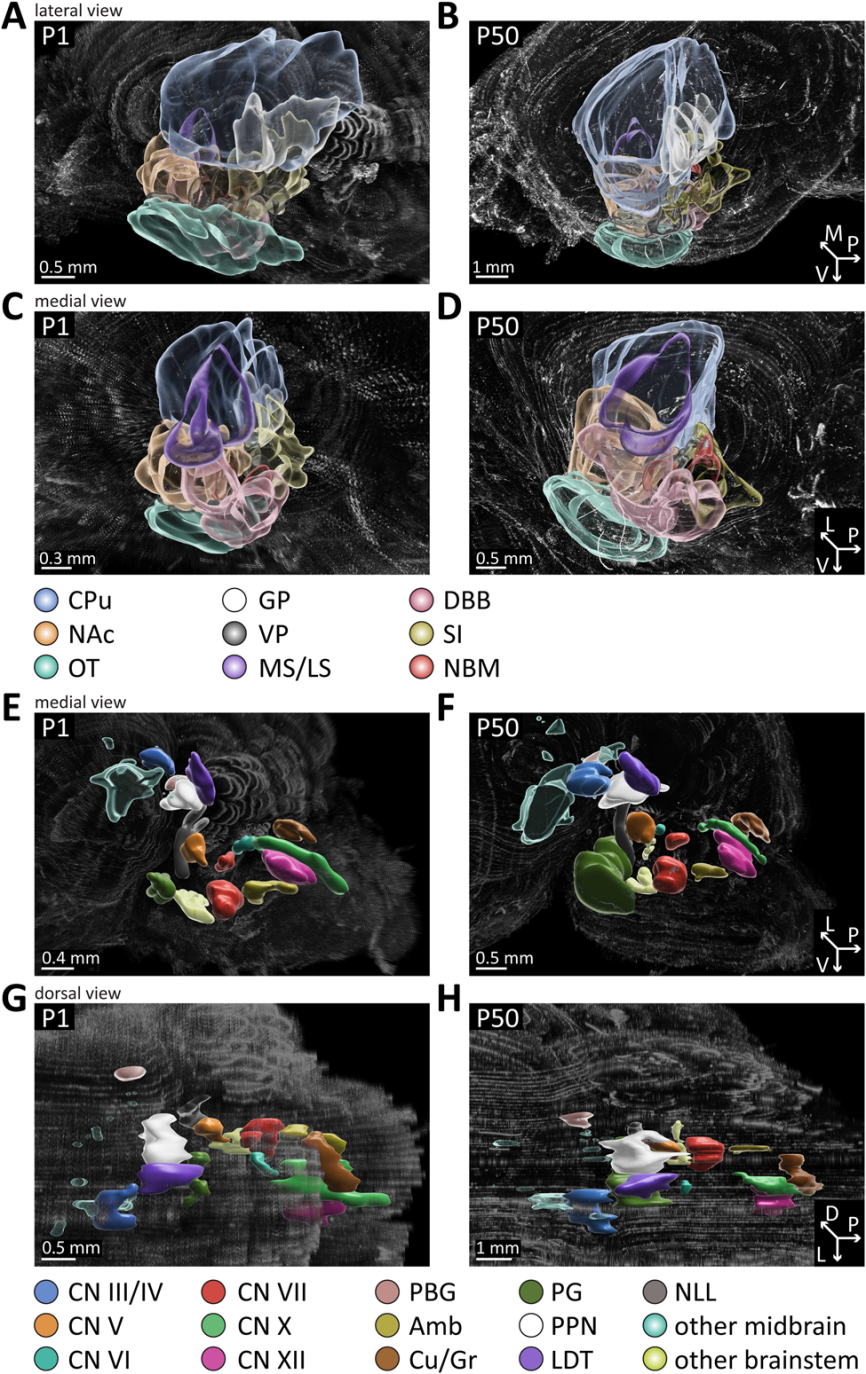
Gross morphology of cholinergic nuclei in the forebrain and caudal brain change little during postnatal development. Reconstructions of serial sectioned brains collected at P1 and P50 were assessed for changes in individual nuclei in the forebrain and caudal brain during development. For better visibility, ACh populations were color-coded by individual brain nuclei in one hemisphere. ***A–D***, Diagram of ACh neurons in forebrain and striatal nuclei (view from outside (***A***, ***B***), view from midline (***C***, ***D***). ***E–H***, Diagram of ACh neurons in the midbrain, pons, and medulla [view from outside (***E***, ***F***), view from midline (***G***, ***H***)]. Abbreviations: CPu, caudate putamen; NAc, nucleus accumbens; OT, olfactory tubercle; GP, globus pallidus; VP, ventral pallidum; MS/LS, medial/lateral septal nuclei; DBB, diagonal band of Broca; NBM, nucleus basalis of Meynert; SI, substantia innominata; CN III/IV, cranial nerve III/IV; CN V, cranial nerve V; CN VI, cranial nerve VI; CN VII, cranial nerve VII; CN X, cranial nerve X; CN XII, cranial nerve XII; PBG, parabigeminal nucleus; Amb, nucleus ambiguus; Cu/Gr, cuneate/gracile nucleus; PG, pontine gray; PPN, pedunculopontine nucleus; LDT, laterodorsal tegmentum; NLL, nucleus of the lateral lemniscus. See Movies 11–12 for rendered view from all sides.

10.1523/ENEURO.0542-23.2024.v11 Movie 11 Animated View at individual cholinergic nuclei in the fore- and hindbrain in P1 brain reconstruction depicted in Fig 6 A, C, E, G. Download Movie 11, MP4 file.

10.1523/ENEURO.0542-23.2024.v12 Movie 12 Animated View at individual cholinergic nuclei in the fore- and hindbrain in P50 brain reconstruction depicted in Fig 6 B, D, F, H. Download Movie 12, MP4 file.

Overall, we identified nine brain anatomical structures containing ACh neuron soma. This includes the spinal cord for E12, E15, and E18. At E12, we detected continuous strains of putative motor and other ACh neurons throughout the entire spinal cord (Fig. 5A, Table 2). At E12, it was not possible to further resolve spinal cord subregions due to the small size of the embryo. At later time points, samples contained very little spinal cord material which prevents a more in-depth analysis of spinal cord ACh neurons. In addition to spinal cord, five of the seven cholinergic CN nuclei in the caudal brain were detected as early as E12, including the hypoglossal (CN XII), the vagus (CN X), the facial (CN VII), the abducens (CN VI), and CN V, but not CN III and CN IV (respectively; Fig. 5*A*). At E15, cholinergic ACh neuron populations in the brain include the remaining caudal brain nuclei, CN III, CN IV, PG, PPN, LDT, and parabigeminal nucleus (PBG), as well as ACh neurons in basal forebrain and striatum (Fig. 5*B*). At E18, additional cholinergic neurons were detected in parts of the hypothalamus. Then at P3, thalamic ACh neurons are consistently present, followed by cerebellar cholinergic innervation at P6 and cortical as well as hippocampal ACh neurons at P9 (Fig. 5*E–H*). Overall, we observed a high reproducibility when ACh neuron populations become cholinergic in all brain anatomical structures using serial sectioned brain reconstruction.

### Gross morphology of ACh neuron nuclei changes little from the neonatal to the mature brain

To further delineate ACh neuron populations at different time points, we identified the major individual nuclei in the forebrain and caudal brain at P1 and P50 according to the Paxinos mouse brain atlas, 4th edition (Paxinos et al., 2020). Overall, we found that all populations present at P50 are already detectable at P1 (Fig. 6*A–H*; Movies 11–12) and change little in their gross morphology during postnatal development.

In the forebrain, we focused on a total of nine ACh neuron-containing subcortical nuclei comprising the striatum and basal forebrain. Despite the brain undergoing massive reorganization and increase in size from P1 to P50, we found that the overall shape and neuron distribution pattern within the forebrain persists throughout this period (Fig. 6*A–D*).

In the caudal brain, we focused on a total of 13 nuclei containing ACh neurons (Fig. 6*E–H*). These 13 structures change very little in their gross anatomy during postnatal development. In addition to these 13 regions, we detected sparse ACh neuron populations in several other nuclei. At P1, it was hard to delineate these nuclei due to size and shape of the brain as well as the sparse distribution of ACh neurons. Therefore, we first identified potential structures at P50 and afterward compared ACh neuron localization at P50 with P1. In the brainstem, we found these additional putative structures: superior olivary complex and inferior olivary complex. In the midbrain, we identified these structures containing ACh neurons: Edinger–Westphal nucleus, periaqueductal gray, mesencephalic reticular nucleus, substantia nigra, ventral tegmental area, red nucleus, and inferior colliculus.

Taken together, these data indicate that the presence and gross anatomy of populations of ACh neuron soma in the forebrain and caudal brain changes little during postnatal development.

## Discussion

Here, we report a dataset on the development of ACh neurons. By employing serial sectioned brain reconstruction, we studied the distribution of tdTomato-labeled ACh neurons in transgenic mice. Firstly, our data provide novel insights into the timeline of when ACh neurons become cholinergic. We report additional or revised ACh neuron populations in the brainstem, the midbrain, and the hypothalamus compared with those previously described in other recent studies (Li et al., 2018; Gamage et al., 2023). Secondly, we describe the changes in cholinergic innervation of the cerebellum as well as the cholinergic projections from the habenula to the hindbrain. Thirdly, we outline the order of cholinergic innervation of the sensory cortex layers, prefrontal cortex layers, hippocampus, and dorsal striatum.

### When are ACh neurons becoming cholinergic?

ACh neurons and nAChRs can be detected from an early time point in the embryonic brain but the findings vary based on the experimental conditions (Cimino, 1995; Jaarsma et al., 1997; Zhang, 1998; Abreu-Villaca et al., 2011; Zhang et al., 2016; Broide et al., 2019; Abbasi et al., 2020; Alzu’bi et al., 2020; Colangelo et al., 2022; Saad et al., 2022). While it has been shown that hindbrain CN neurons are born approximately at E9, basal forebrain ACh neuron between E10 and E13.5, and neurons in the habenula between E14 and E18, the date of birth does not explain when ACh neurons become cholinergic (Finlay and Darlington, 1995; Allaway et al., 2020; Ananth et al., 2023; Funato et al., 2000). Based on our data, there are three major time windows during which ACh neurons become cholinergic: (1) before E12, for ACh neurons in the spinal cord and in the brainstem cranial nerves; (2) between E12 and E15, for ACh neurons in the striatum, basal forebrain, and midbrain; and (3) after E15, for ACh neurons in the hypothalamus, thalamus, hippocampus, and cerebral cortex.

According to our data, ACh neurons becoming cholinergic is highly synchronized between individual cholinergic nuclei located in the same brain anatomical structures and approximately follows the caudorostral direction in which the brain is formed.

During embryonic development, the eight individual parts of the brainstem, called rhombomeres, form from neural crest cells located between the notochord and epidermis (Mendez-Maldonado et al., 2020). A unique combination of several homeobox proteins and other transcription factors define the molecular identity and function of neurons along the rostrocaudal and dorsoventral axis that form during rhombomere development (Gray, 2013; Mendez-Maldonado et al., 2020). Most rhombomeres contain one or even two CNs, which may explain the variability in neuron morphology between the different nuclei in the brainstem.

Similar mechanisms lead to the fate decisions of midbrain and forebrain neurons at different times during development. Forebrain ACh neurons arise from all proliferation areas in the subpallium except for the lateral ganglionic eminence (Ahmed et al., 2019). Within the embryonic subpallium exist subdomains divided by individual transcription factor expression patterns (Flames et al., 2007). From these subdomains, populations of molecularly discrete progenitors of ACh neurons differentially migrate to the target regions, such as the individual basal forebrain nuclei (Ahmed et al., 2019; Ananth et al., 2023). While there is experimental data on how basal forebrain ACh neuron projections target different brain regions and how individual ACh neuron subpopulations within the same brain region fulfill distinct functions (Li et al., 2018; Kim et al., 2024), more research is needed to combine molecular identity with projection pattern and circuit function. Here, we report that ACh neurons in the basal forebrain and striatum start to become cholinergic around the same time shortly before E15, regardless of molecular identity and birth date of the progenitor in the subpallium. Later during development, other ACh neuron nuclei become cholinergic after E18 or postnatally, including parts of the epithalamus, and caudal hypothalamus parts. Since homeobox proteins work in concert with other brain region-specific and ubiquitous transcription factors, our data suggests that activation of cholinergic gene expression is possibly triggered as the neurons migrate to or arrive at their destination (Cho et al., 2014; Ananth et al., 2023). Hindbrain CN nuclei projecting to the periphery likely play a vital role in development of craniofacial shape and are already cholinergic at E12, while the forebrain or cerebellum remains still in formation (Sudiwala and Knox, 2019). Further studies are required to examine the mechanisms that guide the time point when ACh neurons become cholinergic and how cholinergic signaling contributes to maturation of the target brain region.

ACh neurons in the cortex are a subpopulation of VIP interneurons, implicated in specific functions such as long-term attention or additional inhibitory drive (Li et al., 2018; Obermayer et al., 2019; Dudai et al., 2020; Granger et al., 2020). Although VIP expression can be detected in the brain as early as E14, we did not detect any cortical ACh interneurons until after P6 (Waschek et al., 1996). It can, therefore, be assumed that cortical ACh neurons are potentially GABAergic VIP interneurons before they become cholinergic. Given that VIP/ChAT neurons express little ChAT and few cortical neurons respond to VIP/ChAT neuron stimulation with nAChR postsynaptic potentials in the mature mouse brain, the exact role of ACh interneurons in cortical function has yet to be determined (Obermayer et al., 2019; Dudai et al., 2020; Granger et al., 2020). However, a developmental role is possible as the onset of cortical ACh neurons overlaps with the apoptotic phase between P4 and P7, as well as the onset of astrocyte maturation (Duan et al., 2020; Clavreul et al., 2019). Concomitantly, basal forebrain ACh neurons show a high coexpression of GABAergic markers during late embryonic and early postnatal development (Granger et al., 2023; Lozovaya et al., 2024). Furthermore, striatal ACh neurons require GABAergic excitation during the first postnatal week for adequate striatal circuit function (Lozovaya et al., 2023). Since the spatial distribution of GABAergic synapse markers in cholinergic neurons throughout development is unknown, further studies need to examine the role of GABA cotransmission from ACh neurons in neural circuit formation and refinement.

### When are brain regions innervated by cholinergic fibers?

We observed that cholinergic fibers innervate various brain regions in a stepwise manner. These steps include the following: (1) the arrival of cholinergic fibers in layers or subregions; (2) the progressing innervation (all regions studied) and activation of cholinergic gene expression in local ACh interneurons (hippocampus and cortex only); (3) the formation of a more complex synaptic structure (cerebellum only); and (4) the refinement and/or compartmentalization of cholinergic innervation.

Our findings on changes of cholinergic innervation during development in the hippocampus, striatum, and cerebellum are consistent with the literature (Matthews et al., 1974; Clos et al., 1989; Aznavour et al., 2003; Aznavour et al., 2005; Zhang et al., 2016; Fore et al., 2020). However, according to our data, L6 and L1 of the barrel field in the primary somatosensory cortex are innervated first by cholinergic neurons, while L2–5 remain sparsely innervated by fibers passing through to L1. The L2–5 gap in the cholinergic innervation is later filled and its distribution is subsequently refined. Yet, this L2–5 gap in cholinergic innervation is surprising because cholinergic neurons which predominantly project to L2–4 are born before those innervating L1 (Allaway et al., 2020). Thus, the birthdate is unlikely a predictor when cortical innervation occurs. However, we found an exception for prefrontal areas where a late population of fibers is added to L1 between P12 and P50. Furthermore, while the innervation pathway (septal vs rostromedial) is a predictor for which layers are innervated (Bloem et al., 2014; Allaway et al., 2020), it does not predict when and in which order specific cortical layers become innervated. While this data also confirms that cholinergic innervation of the cortical layers occurs from inside out like the brain itself (Angevine and Sidman, 1961), the growth of cholinergic innervations is likely defined by layer-dependent needs. One explanation for this could be that cholinergic innervation up to the top of L1 needs to be established through neurotrophic or other signaling pathways before innervation of intermediate layers can be established (Boskovic et al., 2018; Boskovic et al., 2019; Ananth et al., 2023’ Thomasen et al., 2023). Further studies are required to study the mechanisms of cholinergic innervation of the cortex.

We also observed that the activation of cholinergic genes in cortical interneurons coincides with innervation of cortical layers L2–4, where most of the cholinergic interneurons reside (Li et al., 2018). This potentially means that there are mechanisms which coordinate the cholinergic drive between projection and local interneurons. Taken together, this dataset provides insights about cholinergic signaling onset throughout the brain. This is critically important to understand the role of ACh neurons in brain development, neurotoxicology mechanisms in response to environmental exposure, as well as disease mechanisms associated with cholinergic signaling.

### Experimental limitations

Consistent with previous work, we found that the expression of the ACh neuron marker ChAT changes over time or is low in specific brain regions, such as the lateral septum, cerebral cortex, or cerebellum (Clos et al., 1989; Li et al., 2018; Gamage et al., 2023). Therefore, the well-characterized ChAT-IRES-Cre driver mouse line was used in combination with Cre reporter lines to express tdTomato in ACh neurons to ensure robust soma detection. In the prenatal brain, additional immunostaining was required to enhance the tdTomato signal in the forebrain. Another caveat is that the ChAT-IRES-Cre animal model we used still contains a partial Neo cassette which may cause off-target effects. Although we did not detect any ectopic Cre expression in our experiments, we cannot rule out off-target effects of Cre expression or the partial Neo cassette. Furthermore, Cre-mediated recombination does not instantly induce transgene expression and yield robust fluorescent signals (Metzger and Chambon, 2001). This is why our findings could potentially be inaccurate by 1 or 2 d due to delayed transgene expression. Therefore, we chose time intervals of at least 2 d between the individual sectioning time points to account for this caveat.

Moreover, once the Cre driver line removes the double-floxed Stop codon from the Rosa26 locus, tdTomato expression is switched on but cannot be switched off anymore. Therefore, our data shows all neurons which were at one point cholinergic but not necessarily are cholinergic anymore. Especially those ACh neuron populations with low ChAT expression may not strictly be cholinergic anymore as they do not release ACh (Granger et al., 2020; Hunt et al., 2022). Likewise, Cre-induced recombination is not always successful, which is why only ∼70–95% of ACh neurons are tdTomato positive.

Lastly, given the extensive arborization of ACh neuron projections, our experimental strategy is unsuited to dissect developmental changes of cholinergic innervation originating from different regions or individual neurons (Wu et al., 2014; Allaway et al., 2020).

### Future directions

Further steps to investigate the cholinergic system on a whole-brain level should be aimed at combining techniques or datasets to achieve a more comprehensive understanding of processes happening during brain development. The role of cholinergic signaling needs to be clarified during the different stages of neural circuit formation (Lim et al., 2018). These need to include input–output relationships of ACh neuron populations and how these connections are formed (Gielow and Zaborszky, 2017). Additionally, it is essential to study the location and composition of cholinergic synapses, nicotinic, as well as muscarinic receptors at different developmental time points similar to recent research at the neuromuscular junction (York and Zheng, 2017; Badawi and Nishimune, 2018). Besides, the development of the cholinergic system should be assessed in relationship with other neurotransmitter or neuromodulator systems on a whole-brain level (Colangelo et al., 2022). Lastly, ACh neurons coexpress GABAergic markers during development, and GABA cotransmission is important for mature brain function (von Bartheld and Rubel, 1989; Goral et al., 2022; Granger et al., 2023; Lozovaya et al., 2023). Therefore, more research is required to better understand the role of corelease and cotransmission of protons, nucleic acids, ions, and other neurotransmitters during neural circuit formation (Burnstock, 1976; Upmanyu et al., 2022; Nelson et al., 2014). Since synaptic protein translation often happens directly at the synapse, bulk or single-cell RNA sequencing techniques may not provide meaningful data on composition and heterogeneity of cholinergic synapses (Hafner et al., 2019). Consequently, future research needs to address these issues by exclusively investigating isolated synaptic mRNA or proteins.

Given the large differences in size, lifespan, and cranial complexity between mice and humans, ultimately other model organisms need to be studied to better assess the translatability of findings from rodent research in brain development (Wallace and Pollen, 2023; Wildenberg et al., 2023; Moreno et al., 2009; Pauly et al., 2013).

## Synthesis

Reviewing Editor: Benjamin Deneen, Baylor College of Medicine Decisions are customarily a result of the Reviewing Editor and the peer reviewers coming together and discussing their recommendations until a consensus is reached. When revisions are invited, a fact-based synthesis statement explaining their decision and outlining what is needed to prepare a revision will be listed below. The following reviewer(s) agreed to reveal their identity: Robert Machold.

The reviewers find the manuscript to be of interest and noteworthy. Despite the acknowledgment of these merits, there are several substantive issues that they highlight that need to be addressed before this paper is suitable for publication. In terms of the general manuscript, the overall organization of the manuscript needs to be improved, with the inclusion of subheadings, etc. Also in the the discussion, more context of how these findings fit into the broader field needs to be conveyed. Experimentally, the 3D models need to be refined to provide more resolution, this was considered a weak point in the manuscript. Additional immunostating was suggested, along with a consideration of sex difference, since that data has already been captured. Finally, caveats concerning the mouse lines need to be addressed and the experimental limitations also need to be raised.

## Author Response

Manuscript Instructions --------------------------------------------- Synthesis of Reviews:

Computational Neuroscience Model Code Accessibility Comments for Author (Required):

N/A Synthesis Statement for Author (Required):

The reviewers find the manuscript to be of interest and noteworthy. Despite the acknowledgment of these merits, there are several substantive issues that they highlight that need to be addressed before this paper is suitable for publication.

1) In terms of the general manuscript, the overall organization of the manuscript needs to be improved, with the inclusion of subheadings, etc.

2) Also in the discussion, more context of how these findings fit into the broader field needs to be conveyed.

3) Experimentally, the 3D models need to be refined to provide more resolution, this was considered a weak point in the manuscript.

4) Additional immunostaining was suggested, along with a consideration of sex difference, since that data has already been captured.

5) Finally, caveats concerning the mouse lines need to be addressed and the experimental limitations also need to be raised.

Reviewer 1 The manuscript entitled "Acetylchloline neurons become cholinergic during three time windows in the developing mouse brain" (eN-NWR-0543-23) presents a map of the developmental emergence of cholinergic neurons throughout the brain using genetic labeling. While the developmental trajectory of ChAT expression in different brain regions has been documented previously, the ChAT-ires-Cre; tdTomato reporter model the authors employ provides a consistent readout of ChAT neurons across the various developmental stages examined in this study. The finding that ChAT neurons within each nuclei or brain region reach maturation (as defined by ChAT expression) at similar times regardless of some variation in birthdate is noteworthy.

Specific comments:

6) The ChAT-ires-Cre line used by the authors (Jax #006410) was submitted to Jax by Dr. Bradford Lowell over a decade ago, and still harbors the frt-neo-frt cassette used for positive selection of targeted ESC clones during the generation of this line. While I don't think this greatly impacts the overall findings of this study, the authors should note that a newer version of this ChAT-ires-Cre line with the neo cassette removed is now available at Jax (#031661). Jax reports that there can be ectopic Cre-mediated recombination in the original (neo+) line, so perhaps the authors can add a brief discussion of this possibility, and whether they ever observed any animals with clear ectopic reporter expression with their ChAT-ires-Cre; Ai9 or Ai14 crosses. In addition, it would be useful for the authors to consider this caveat when discussing the (mostly subtle) differences between tdTomato labeling and ChAT IHC neurons they observe.

We thank you for the comment. In fact, we checked the Jackson Laboratories website for the animal model you proposed without Neo cassette (B6.129S-Chattm1(cre)Lowl/MwarJ, RRID:IMSR_JAX:031661). The description is not entirely clear about whether the partial Neo cassette was removed. The following is written on the Jackson Laboratories website (https://www.jax.org/strain/031661#): "In 2018, The Jackson Laboratory Repository bred some Stock No. 028861 animals to C57BL/6J-congenic ROSA26::FLPe strain (Stock No. 009086) to facilitate germline removal of the neo cassette. The resulting animals with the ChAT-IRES-Cre::SV40pA::Δneo knock-in allele, Chattm1(cre)Lowl, were bred together or to wildtype (and the ROSA26::FLPe was removed) to establish this C57BL/6J-congenic ChAT-IRES-Cre::SV40pA::Δneo colony as Stock No. 031661.

Characterization performed at The Jackson Laboratory (2019-2020) determined there is an incomplete neo sequence (224 bp) located 310 bp downstream of Cre STOP codon and 70 bp upstream of SV40 polyA sequence." It was, therefore, not entirely clear to us whether this new animal model is truly Neo cassette-free.

However, in our lab we have been using the strain Jax #006410 for more than a decade and did not observe any ectopic Cre expression in our experiments.

A similar statement has been added to the Discussion under "Experimental Limitations".

7) In the "Experimental Limitations" section, the authors state that "tdTomato expression from the Rosa26 locus is not strong enough to observe projections from the basal forebrain to the cortex or the hippocampus" but this statement is not accurate. First, it should be mentioned that Ai9 and Ai14 (essentially the same reporter, but Ai9 retains the neo selection cassette) are reporters that although located within the Rosa26 locus, use an internal CAG promoter and other features (WPRE) to drive high levels of tdTomato expression. With antibody enhancement of the tdTomato signal (e.g., with rabbit anti-RFP, Rockland #600-401-379), ChAT axons in cortex and hippocampus can be readily visualized with Ai9, Ai14, or Ai65 (cre+flp tdTomato reporter; used in Allaway et al., 2020).

We thank you for this comment and want to assure you that we are aware of this paper. However, we admit that our statement may have been written in a confusing way. Initially, we wanted to say, that cortical cholinergic inputs originate from different brain regions, and we cannot distinguish which fibers originate from which region using our experimental approach; not even if the signal was enhanced by immunostaining. Since there are developmental differences in cholinergic nuclei, such a distinction may be important to better understand temporal changes in cortical or hippocampal development. An approach of labeling single neurons, like what has been used in Wu et al. eLife 2014, or viral injections may be helpful to perform this kind of separation.

To further address this comment, we stained slices for DsRed (using the antibody proposed by you) at different timepoints to assess the organization of cholinergic fibers during development. We found a generally increased cholinergic innervation as well as an improved compartmentalization in various brain regions during the postnatal period. We added this data to the manuscript as new Fig 4.

The new data is consistent with previous literature, such as Allaway et al. 2020 and others we cite in the discussion, as it supports a stepwise innervation of cortical regions starting with deeper layers. At P3, only the cortical layers 1 and 6 are innervated. At P6, cholinergic fibers are only passing through L2-4 to L1 but not branching off. However, L1 is strongly innervated before L2-4 which is a novel and surprising finding in this manuscript. Furthermore, this data shows that there is synchrony between innervation of superficial cortical layers and the activation of cholinergic genes in cortical and hippocampal ACh interneurons. To our knowledge, this is the first time that this synchrony has been reported, although the mechanism underlying this synchrony has yet to be determined. However, this may provide a hint when cholinergic interneurons start to play a role in cortical/hippocampal circuit function. While the prime target for cortical ACh interneurons is layer 1, these interneurons mostly provide inhibitory drive to few cells in the mature brain (Obermayer Nat Commun 2019, Granger eLife 2020, Dudai Plos Biol 2020). It is possible that the role of cortical ACh interneurons may rather be critical during development, as it was previously described for striatal ACh interneurons which provide excitatory GABAergic drive in the immature dorsal striatum. This is consistent with findings that large numbers of cholinergic neurons in the basal forebrain express levels of GABAergic markers (GAD1/2, Slc32a1) during the second of postnatal development (Granger Front Neur Circ 2023). It is our goal to further elucidate the mechanism during future studies.

Reviewer 2 Despite recent advancements, the comprehensive assessment of developmental changes in cholinergic (ACh) neurons has been limited. This paper aimed to clarify when ACh neuron populations become cholinergic in the mouse brain during development. The authors crossbreed homozygous ChAT-IRES-Cre females with homozygous male Ai9 or Ai14 reporter mice and process serial brain sections at several pre- (E,12,E15,E18) and postnatal (P1,P3,P6,P9, P12,P21,P50 and 1 year) time points. They suggest three crucial time windows, and apparently neurons becoming cholinergic within individual nuclei and anatomical regions in the mouse brain with a high synchrony shortly after birth, before major sensory processes begin.

Major concerns:

8) While, the processing for histology and 3D rendering of many brains is a major effort, without quantitative data and statistics, it is difficult to validate the results. The verification of labeling efficiency by comparing tdTomato-positive cells with the ACh neuron marker ChAT is an important process. As the manuscript states, Cre-induced recombination is not always successful and the authors mention that 75-80% of basal forebrain ACh neurons are tdTomato positive, though it is unclear how they arrived to this conclusion. The authors state 'Ach neuron marker ChAT overlaps with tdTomato in many but not all brain regions (Title of Extended Data Figure 1, lines 701-702). More information on the percentage of overlap or quantification of labeling efficiency would add depth to the validation process, though they characterize the work with 'a high labeling efficiency' (line 172).

We agree with you that it is vitally important to verify the validity of our findings. To address this comment, we counted about 15000 cells in 300 z-stacks of various brain regions. We found that all cholinergic nuclei in the fore-, mid-, and hindbrain had more than 70% of tdTomato-positive ChAT cells. We added the results in Fig 1-1 I.

9) The study mentions some comparisons with existing literature (e.g., AChE and ChAT expression patterns in the rat brain). Providing more context or discussing how these findings align or diverge from previous research could strengthen the discussion. 'This somewhat resembles AChE and ChAT expression patterns in rat brain as well fluorescent marker expression in Ach projections in mice..." is rather vague. At other places it reads 'Ach neurons are unevenly distributed in different cortical areas" (line 215). How differently? Although Table 2 list various number pairs for brain regions and time points, it is unclear what the numbers represent. Please provide exact number of animals for each cohorts.

We agree with you, that this was not the most elegant way to compare our results with previous literature. In the original manuscript, it was our main concern to adhere to the space limitations and so we kept everything as brief as possible. In our revision, however, we added Fig 4 where we studied the cholinergic innervation of various brain regions between P6 and P50. Therefore, we transferred the comparisons to the part of the discussion which addresses Fig 4. We cited further literature about the other brain regions for completeness.

10) It is difficult to accept the view that 'birth of Ach neurons and initiation of cholinergic gene expression is 'highly coordinated' (line 17-18) and 'highly synchronized by brain anatomical structure' (line 27) in the lack of quantitative data and lumping together cholinergic neurons, including striatal ACh interneurons, basal forebrain corticopetal and brainstem-spinal cord motor neurons with a diversity of functions and developmental origins. In fact, the authors, cannot decide whether 'activation of cholinergic gene expression could be triggered through one universal or several independent but synchronous mechanism' (lines 317-318).

We agree that this conclusion is too simple. Due to space constraints, we did not elaborate further in the original discussion. We addressed this comment in part by our response to point #8. While our added data at E15 and E18 (Fig 1) indicates that ACh neurons are cholinergic at their destination (e. g. in the dorsal striatum or basal forebrain) but not in the proliferation zones at E15, we cannot rule out that they are cholinergic before leaving the proliferation zone during an earlier timepoint. Additionally, we noticed that the highest density of tdTomato-positive ACh neurons in the ventrocaudal half of the dorsal striatum. Therefore, we also adjusted the discussion accordingly.

Other concerns 11) Since both sex was collected, did the authors find any sex differences in the development of the cholinergic system? We added Extended Data Table 1 to include information on the animal sex. As you can see there, most developmental time points have animals of both sexes. In our experiments, the cholinergic innervation of females and males are virtually indistinguishable.

12) Reorganizing documentation using 3D rendering (Figure 3, 4, Extended Data Figures 2,3) could help in presenting complex information more clearly. Since brain nuclei are not labeled individually, without some fiducial markers, the images are hard to comprehend.

We agree that the small images are not sufficient to illustrate the three-dimensional datasets. Therefore, we generated 12 short animated videos for every age for Fig 5 and Fig 6, which we uploaded with the revised manuscript.

13) While counterstaining of ChAT was carried out, would it be possible to carry out a NeuN counterstaining as well, especially during the embryonic stages? We followed your advice and added counterstainings for NeuN for ages from E15 to P12. These NeuN stains show us that the organization of mature neurons changes postnatally depending on the brain region. According to our data, these changes in NeuN staining are mostly done by P6, except for the cortex. All NeuN stains for various brain regions were added to the Extended Data as Fig 1-2, 1-3, 4-1, 4-2, 4-3.

14) The manuscript may benefit from a clearer organization. Breaking down the information into subsections or using more subheadings could enhance readability. Why not start the results with the embryonic expression (following chronological order of development)? We followed your advice and reorganized the manuscript. Firstly, we added additional subsections. Secondly, we begin the Results section by verification of the animal model. Afterwards, we assess the embryonic development before we assess the postnatal development. After the assessment of soma locations, we assess the changes in cholinergic innervation during development. In the end, we discuss the 3D reconstructions.

15) The description of the localization of the postnatal cholinergic neurons is not very novel and it's not clearly communicated if the authors have found any changes in either the localization or numbers.

Thank you for the comment. In the original manuscript version, we mentioned our new findings in the postnatal brain in the very beginning of our discussion. In contrast to other recent publications on ACh neurons populations in the brain (Li et al. PNAS 2017, Gamage et al BMC Neurosci 2023), we found additional cholinergic neurons particularly in other parts of the hindbrain, such as the nucleus of the lateral lemniscus and the cuneate/gracile nucleus, and in the lateral septum in the forebrain. However, we improved the wording, as it was not clearly communicated. In the revised version of the manuscript we added data, which indicates synchrony between the cholinergic innervation of brain regions, such as the cortex and hippocampus, and the activation of cholinergic gene expression in local interneurons. Since the role of cortical cholinergic interneurons in the mature brain is unclear and these interneurons express little ChAT, it is possible that they predominantly play a role during development.

Taken together, we believe that our new data strengthens our findings as well as the quality of the manuscript, while also providing novel findings on cholinergic innervation in the postnatal mouse brain.

16) Results section should not contain references to other articles, they belongs to discussion (lines 189, 212).

Since we rewrote some of our discussion, we removed the references from the results and moved them to the discussion parts where they fit best.
