# Supplementary material for: Acetylcholine Neurons Become Cholinergic during Three Time Windows in the Developing Mouse Brain
Source: eNeuro. 2024 Jul 11;11(7):ENEURO.0542-23.2024. doi: 10.1523/ENEURO.0542-23.2024 (PMC11253243; doi:10.1523/ENEURO.0542-23.2024)
Supplement: Table 1-1 — Animals used for experiments depicted in different figures. Download Table 1-1, DOC file. [file eneuro-11-ENEURO.0542-23.2024-s013.doc]

**Extended Data Table 1: Animals used for experiments depicted in different figures.**

| **Fig 1, Fig 4, Ext. Data Fig 1-6** | | | |  | **Ext. Data Fig 8** | |  | **Ext. Data Fig 9** | | |
| --- | --- | --- | --- | --- | --- | --- | --- | --- | --- | --- |
| **age** | **♂** | **♀** | **N/A** |  | **age** | **sex** |  | **age** | | **sex** |
| **Fig 4, Ext. Data Fig 1** |  |  |  |  | **E12 (A)** |  |  | **P6 (A)** | |  |
| P50 | 2 | 1 | 0 |  | left | ♀ |  | left | | ♀ |
| **Fig 1, Ext. Data Fig 2** |  |  |  |  | mid | ♀ |  | mid | | ♂ |
| E15 | 1 | 3 | 0 |  | right | ♂ |  | right | | ♂ |
| **Fig 1, Ext. Data Fig 3** |  |  |  |  | **E15 (B)** |  |  | **P9 (B)** | |  |
| E18 | 3 | 2 | 2 |  | left | ♂ |  | left | | ♂ |
| **Fig 4, Ext. Data Fig 4** |  |  |  |  | mid | ♂ |  | mid | | ♂ |
| P3 | 1 | 3 | 2 |  | right | ♂ |  | right | | ♀ |
| **Fig 4, Ext. Data Fig 5** |  |  |  |  | **E18 (C)** |  |  | **P12 (C)** | |  |
| P6 | 2 | 0 | 1 |  | top left | ♀ |  | left | | ♀ |
| **Fig 4** |  |  |  |  | top mid | ♀ |  | mid | | ♀ |
| P9 | 4 | 1 | 0 |  | top right | N/A |  | right | | ♂ |
| **Fig 4, Ext. Data Fig 6** |  |  |  |  | bot left | N/A |  | **P50 (D)** | |  |
| P12 | 1 | 2 | 0 |  | bot mid | N/A |  | left | | ♂ |
|  |  |  |  |  | **P1 (D)** |  |  | mid | | ♂ |
|  |  |  |  |  | left | ♂ |  | right | | ♂ |
|  |  |  |  |  | mid | ♂ |  | **>1yr (E)** | |  |
|  |  |  |  |  | right | ♀ |  | left | | ♀ |
|  |  |  |  |  | **P3 (E)** |  |  | mid | | ♂ |
|  |  |  |  |  | left | ♂ |  | right | | ♂ |
|  |  |  |  |  | mid | ♀ |  | |  | |
|  |  |  |  |  | right | ♂ |  | |  | |
