# Supplementary material for: Acetylcholine Neurons Become Cholinergic during Three Time Windows in the Developing Mouse Brain
Source: eNeuro. 2024 Jul 11;11(7):ENEURO.0542-23.2024. doi: 10.1523/ENEURO.0542-23.2024 (PMC11253243; doi:10.1523/ENEURO.0542-23.2024)
Supplement: Table 2-1 — Data table for mean fluorescence intensity for cortical layers at P3, P6, and P9 (refers to Fig 4 and Extended Data Fig 7). Download Table 2-1, DOC file. [file eneuro-11-ENEURO.0542-23.2024-s014.doc]

**Extended Data Table 2: Data table for mean fluorescence intensity for cortical layers at P3, P6, and P9 (refers to Fig 4 and Extended Data Fig 7).**

| **P3** | **mean (AU)** | **σ** | **n =** |  | **P6** | **mean (AU)** | **σ** | **n =** |
| --- | --- | --- | --- | --- | --- | --- | --- | --- |
| L1 | 286.9 | 36.4 | 48 |  | L1 | 550.9 | 187.3 | 27 |
| L2-4 | 255.9 | 21.0 | 48 |  | L2-4 | 432.1 | 115.6 | 27 |
| L5 | 257.2 | 21.0 | 48 |  | L5 | 453.8 | 89.6 | 27 |
| L6 | 285.8 | 30.8 | 48 |  | L6 | 561.6 | 122.4 | 27 |
| **statistics** | **q=** | **p=** |  |  | **statistics** | **q=** | **p=** |  |
| L1 vs. L2-4 | <0.0001 | <0.0001 |  |  | L1 vs. L2-4 | 0.0019 | 0.0075 |  |
| L1 vs. L6 | 0.2604 | 0.8681 | n.s. |  | L1 vs. L6 | 0.151 | 0.8051 | n.s. |
| L1 vs. ctrl_L1 | <0.0001 | <0.0001 |  |  | L1 vs. ctrl_L1 | <0.0001 | <0.0001 |  |
| L2-4 vs. L5 | 0.2435 | 0.7536 | n.s. |  | L2-4 vs. L5 | 0.09 | 0.4455 | n.s. |
| L2-4 vs. L6 | <0.0001 | <0.0001 |  |  | L2-4 vs. L6 | <0.0001 | 0.0002 |  |
| L2-4 vs. ctrl_L2-4 | <0.0001 | <0.0001 |  |  | L2-4 vs. ctrl_L2-4 | <0.0001 | <0.0001 |  |
| L1 vs. L5 | <0.0001 | <0.0001 |  |  | L1 vs. L5 | 0.0046 | 0.02 |  |
| L5 vs. L6 | <0.0001 | <0.0001 |  |  | L5 vs. L6 | 0.0002 | 0.0006 |  |
| L5 vs. ctrl_L5 | <0.0001 | <0.0001 |  |  | L5 vs. ctrl_L5 | <0.0001 | <0.0001 |  |
| L6 vs. ctrl_L6 | <0.0001 | <0.0001 |  |  | L6 vs. ctrl_L6 | <0.0001 | <0.0001 |  |
| **P9** | **mean (AU)** | **σ** | **n=** |  | | | | |
| L1 | 492.8 | 104.1 | 34 |  | | | | |
| L2-4 | 416.3 | 44.1 | 34 |  | | | | |
| L5 | 417.6 | 36.3 | 34 |  | | | | |
| L6 | 471.0 | 46.1 | 34 |  | | | | |
| **statistics** | **q=** | **p=** |  |  | | | | |
| L1 vs. L2-4 | 0.0001 | 0.0003 |  |  | | | | |
| L1 vs. L6 | 0.1081 | 0.2703 | n.s. |  | | | | |
| L1 vs. ctrl_L1 | <0.0001 | <0.0001 |  |  | | | | |
| L2-4 vs. L5 | 0.2681 | 0.8937 | n.s. |  | | | | |
| L2-4 vs. L6 | <0.0001 | <0.0001 |  |  | | | | |
| L2-4 vs. ctrl_L2-4 | <0.0001 | <0.0001 |  |  | | | | |
| L1 vs. L5 | 0.0001 | 0.0003 |  |  | | | | |
| L5 vs. L6 | <0.0001 | <0.0001 |  |  | | | | |
| L5 vs. ctrl_L5 | <0.0001 | <0.0001 |  |  | | | | |
| L6 vs. ctrl_L6 | <0.0001 | <0.0001 |  |  | | | | |
