# Supplementary figures and images for: Acetylcholine Neurons Become Cholinergic during Three Time Windows in the Developing Mouse Brain
Source: eNeuro. 2024 Jul 11;11(7):ENEURO.0542-23.2024. doi: 10.1523/ENEURO.0542-23.2024 (PMC11253243; doi:10.1523/ENEURO.0542-23.2024)

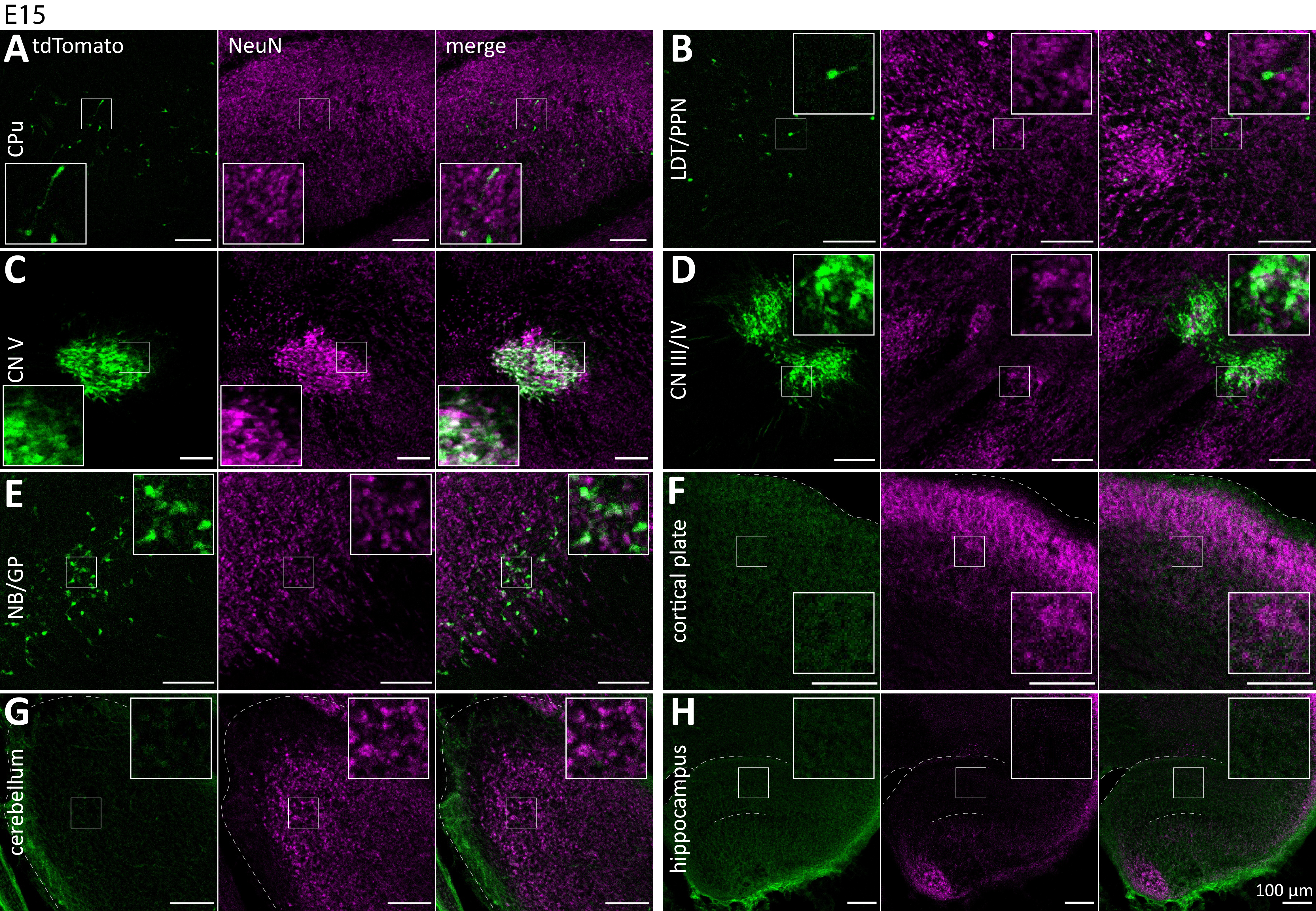

Supplement: Fig 1-2 — Distribution of ACh neurons compared to neuron marker NeuN in the mouse brain at E15. A-H, 50 µm horizontal brain sections of mice expressing tdTomato in ACh neurons at E15 were immunostained for NeuN. Sections were imaged for tdTomato (green) and NeuN (magenta). Brain regions imaged include: CPu (A), LDT/PPN (B), CN V (C), cranial nerve III/IV (CN III/IV, D), NB/GP (E), cortical plate (F), cerebellum (cbl, G), and hippocampus (hc, H). Insets depict magnified ROIs outlined by the small white square. Download Fig 1-2, TIF file. [file eneuro-11-ENEURO.0542-23.2024-s016.tif]

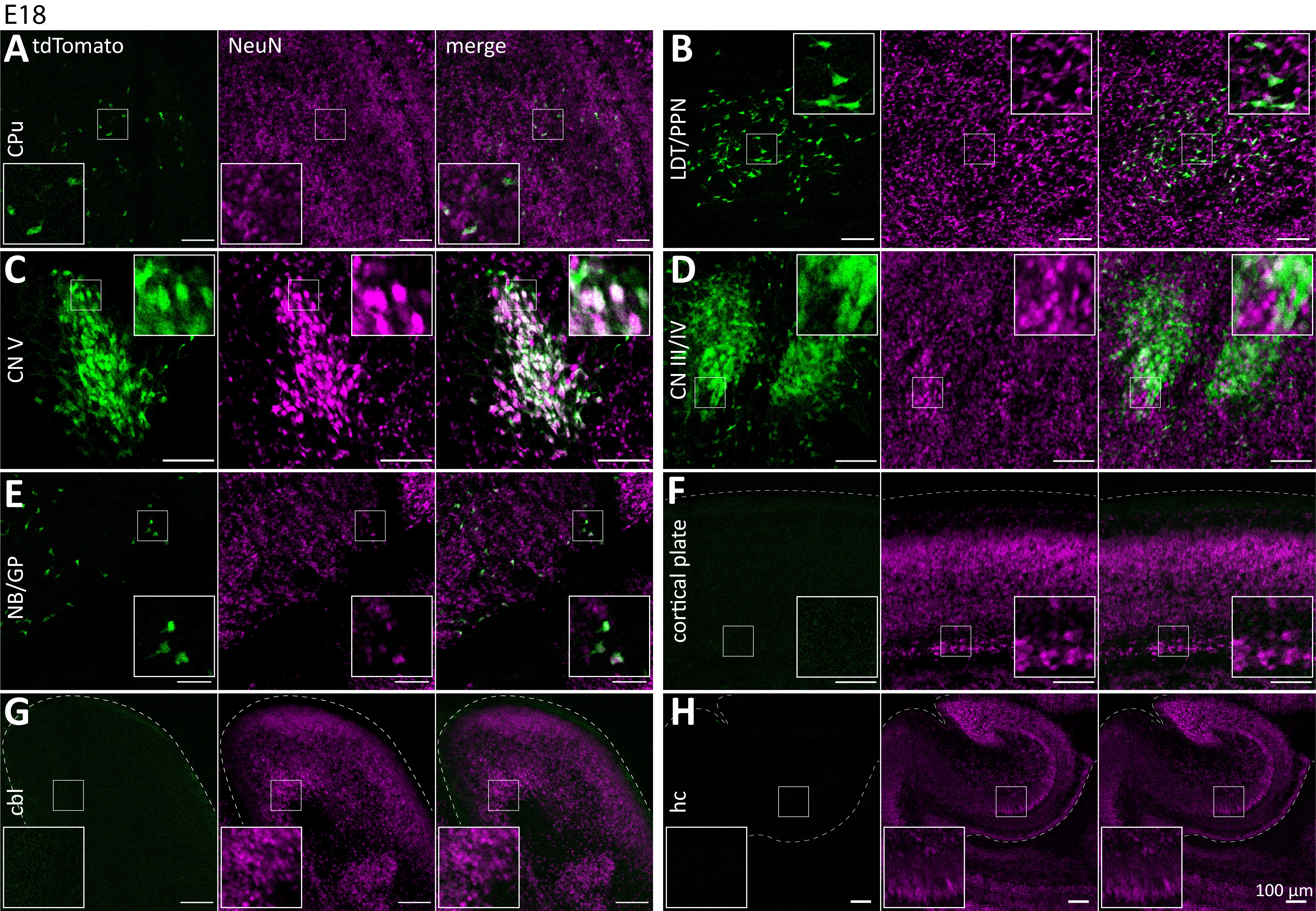

Supplement: Fig 1-3 — Distribution of ACh neurons compared to neuron marker NeuN in the mouse brain at E18. A-H, 50 µm horizontal brain sections of mice expressing tdTomato in ACh neurons at E18 were immunostained for NeuN. Sections were imaged for tdTomato (green) and NeuN (magenta). Brain regions imaged include: CPu (A), LDT/PPN (B), CN V (C), CN III/IV (D), NB/GP (E), cortical plate (F), cerebellum (G), and hippocampus (H). Insets depict magnified ROIs outlined by the small white square. Download Fig 1-3, TIF file. [file eneuro-11-ENEURO.0542-23.2024-s017.tif]

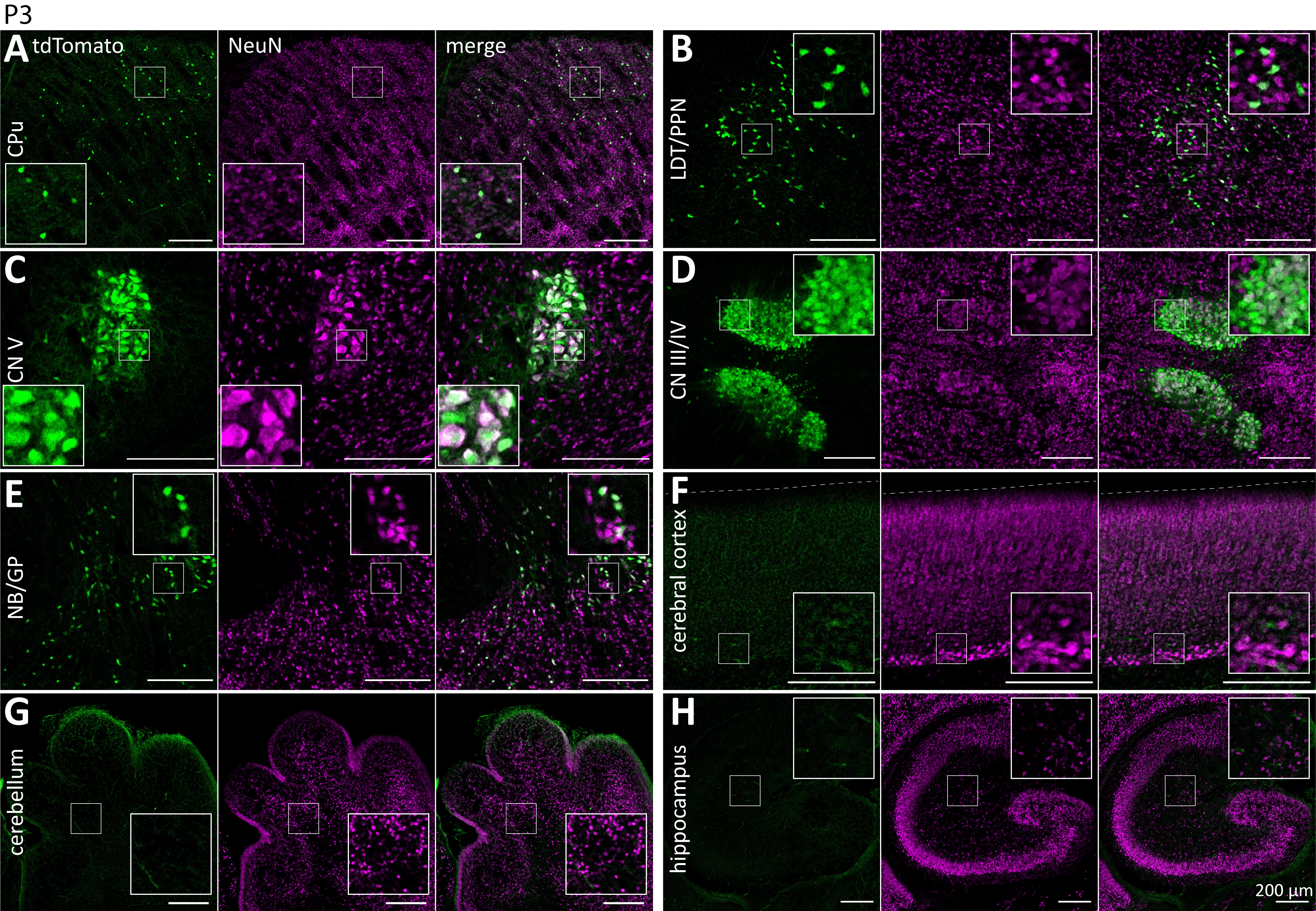

Supplement: Fig 4-1 — Distribution of ACh neurons compared to neuron marker NeuN in the mouse brain at P3. A-H, Brain sections obtained at P3 were assessed as described in Figure 1-2. Imaged brain regions include: CPu (A), LDT/PPN (B), CN V (C), CN III/IV (D), NB/GP (E), S2/AuV (F), cerebellum (G), and hippocampus (H). Insets depict magnified ROIs outlined by the small white square. Download Fig 4-1, TIF file. [file eneuro-11-ENEURO.0542-23.2024-s018.tif]

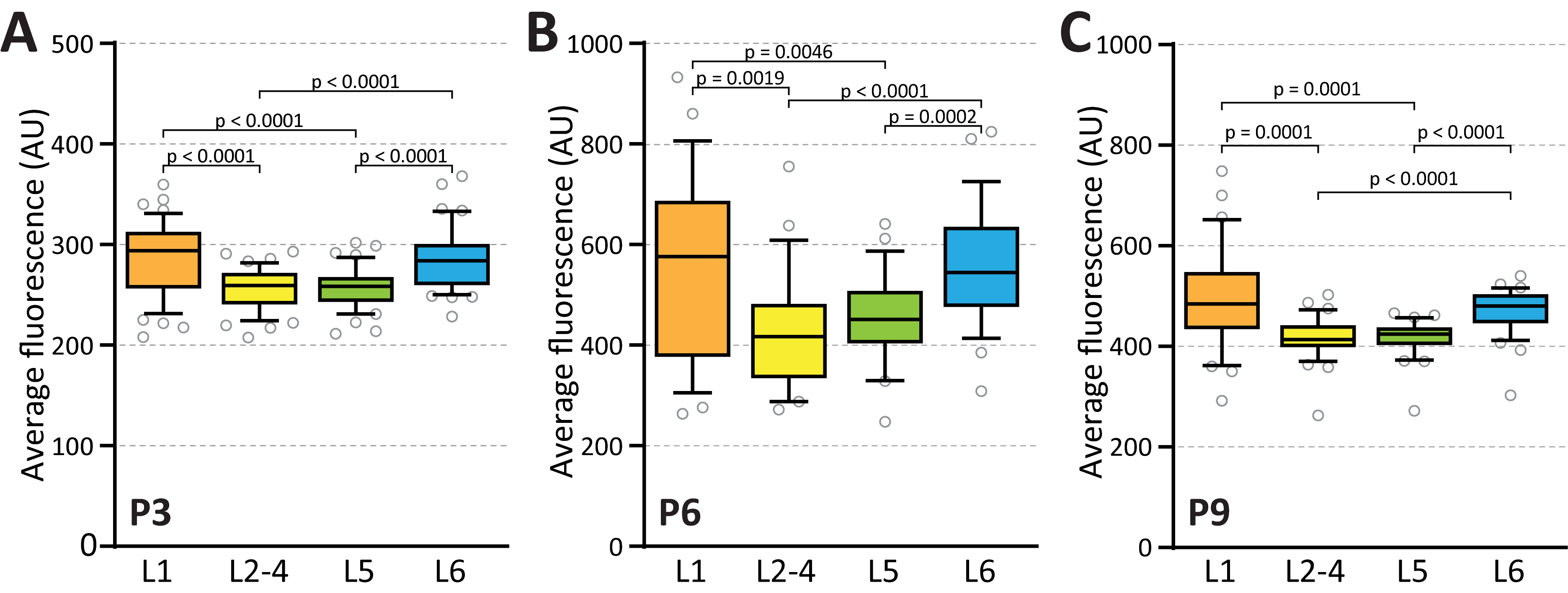

Supplement: Fig 4-4 — Quantification of cholinergic innervation of S1 layers at P3, P6, and P9. A-C, Box plots depicting the average fluorescence intensity as a measure for cholinergic innervation. Cholinergic innervation of S1 L1 (orange) and L6 (blue) is significantly higher than L2-4 (yellow) and L5 (green) from P3-P9. L2-4 and L5 are not significantly different from each other from P3-P9. A Brown-Forsythe and Welch ANOVA with correction for multiple comparisons by controlling the false discovery rate with the Benjamini, Krieger, and Yekutieli method was used to assess differences. Only statistically significant comparisons (q<0.05, p<0.05) are shown. Download Fig 4-4, TIF file. [file eneuro-11-ENEURO.0542-23.2024-s021.tif]

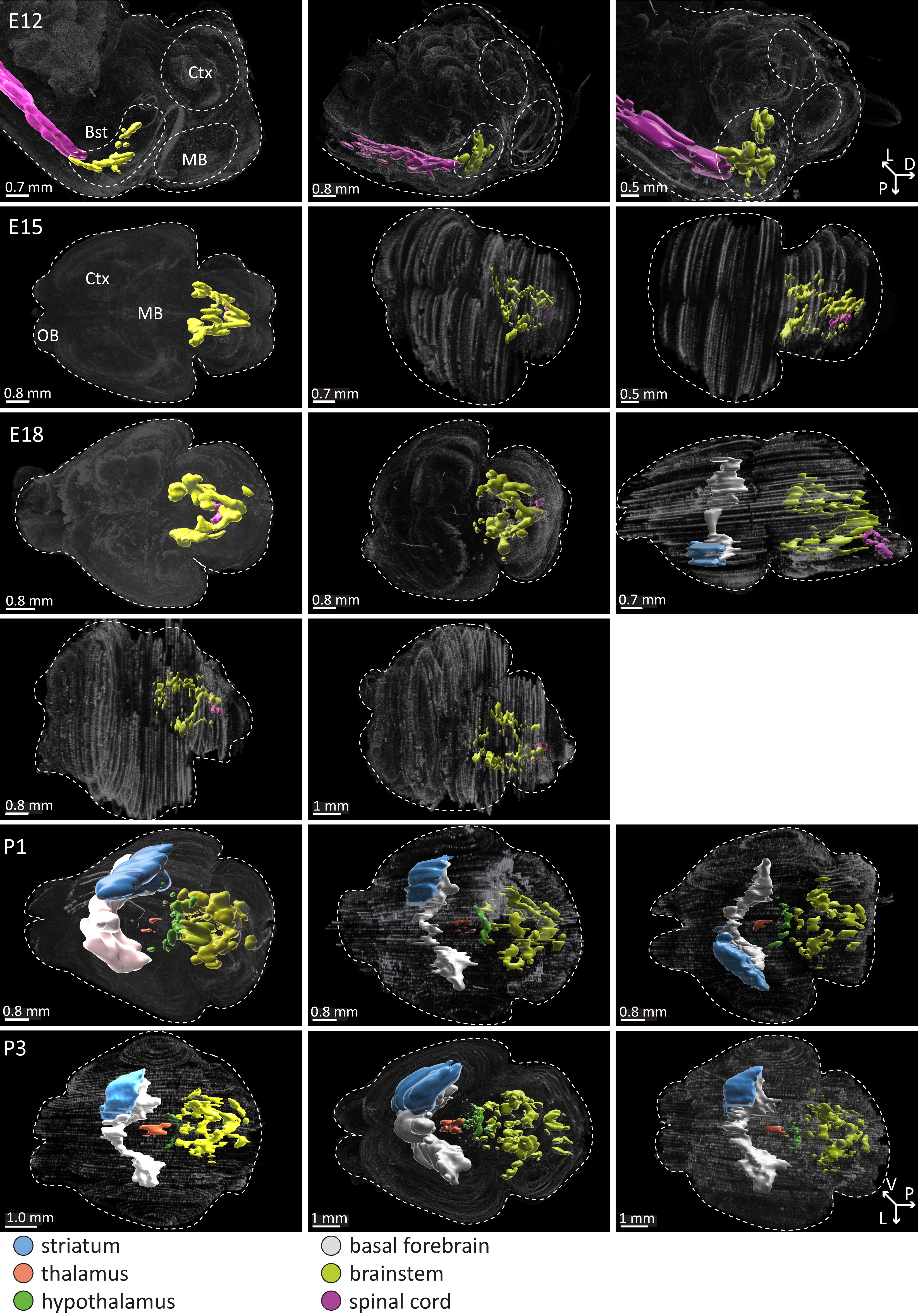

Supplement: Fig 5-1 — All reconstructed mouse brains from E12 to P3. A-E, Reconstructions of serial sectioned brains were collected at E12 (A), E15 (B), E18 (C), P1 (D), P3 (E). For better visibility cartoons of ACh populations were added color-coded by brain regions: transparent dark purple – hippocampus, light blue – striatum, transparent light green – cerebral cortex, orange – thalamus, light gray – basal forebrain, light pink – cerebellum, yellow – caudal brain nuclei, light purple – spinal cord, green - hypothalamus. Download Fig 5-1, TIF file. [file eneuro-11-ENEURO.0542-23.2024-s022.tif]

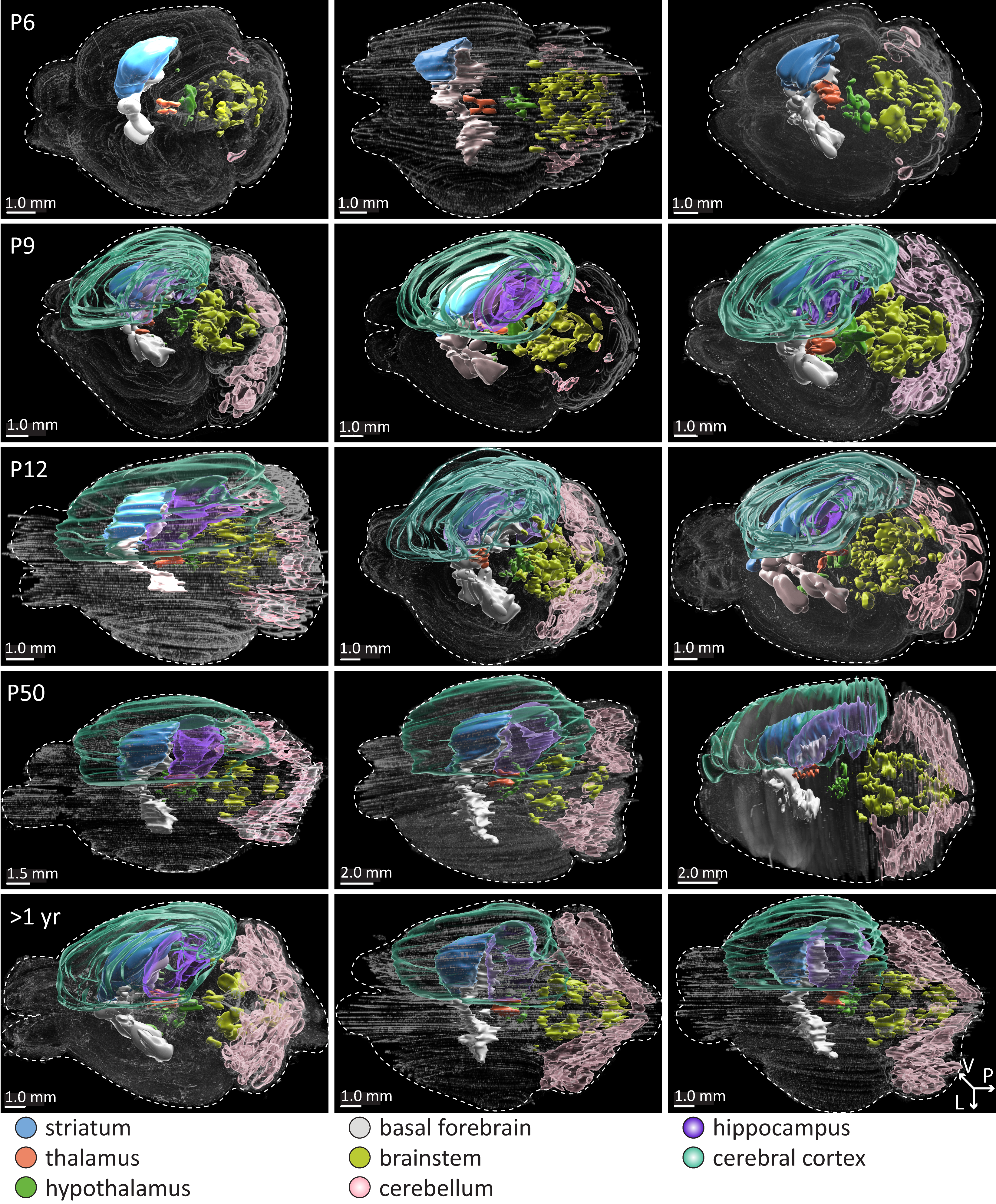

Supplement: Fig 5-2 — All reconstructed mouse brains from P6 to >1 yr. A-E, Reconstructions of serial sectioned brains were collected at P6 (A), P9 (B), P12 (C), P50 (D), and >1 year of age (E). For better visibility cartoons of ACh populations were added color-coded by brain regions: transparent dark purple – hippocampus, light blue – striatum, transparent light green – cerebral cortex, orange – thalamus, light gray – basal forebrain, transparent light pink – cerebellum, yellow – caudal brain nuclei, light purple – spinal cord, green - hypothalamus. Download Fig 5-2, TIF file. [file eneuro-11-ENEURO.0542-23.2024-s023.tif]
